# The Human Blood Metabolome-Transcriptome Interface

**DOI:** 10.1371/journal.pgen.1005274

**Published:** 2015-06-18

**Authors:** Jörg Bartel, Jan Krumsiek, Katharina Schramm, Jerzy Adamski, Christian Gieger, Christian Herder, Maren Carstensen, Annette Peters, Wolfgang Rathmann, Michael Roden, Konstantin Strauch, Karsten Suhre, Gabi Kastenmüller, Holger Prokisch, Fabian J. Theis

**Affiliations:** 1 Institute of Computational Biology, Helmholtz Zentrum München, Neuherberg, Germany; 2 Institute of Human Genetics, Helmholtz Zentrum München, Neuherberg, Germany; 3 Institute of Human Genetics, Technische Universität München, Neuherberg, Germany; 4 Institute of Experimental Genetics, Genome Analysis Center Helmholtz Zentrum München, Neuherberg, Germany; 5 Faculty of Experimental Genetics, Technische Universität München, Freising-Weihenstephan, Germany; 6 German Center for Cardiovascular Disease Research (DZHK e.V.), partner-site Munich, Munich, Germany; 7 Institute of Genetic Epidemiology, Helmholtz Zentrum München, Neuherberg, Germany; 8 Institute of Clinical Diabetology, German Diabetes Center, Leibniz Center for Diabetes Research at Heinrich Heine University Düsseldorf, Düsseldorf, Germany; 9 German Center for Diabetes Research (DZD e.V.), partner-site Düsseldorf, Düsseldorf, Germany; 10 Institute of Epidemiology II, Helmholtz Zentrum München, Neuherberg, Germany; 11 German Center for Cardiovascular Disease Research (DZHK e.V.), partner-site Munich, Munich, Germany; 12 Institute of Biometrics and Epidemiology, German Diabetes Center, Leibniz Center for Diabetes Research at Heinrich Heine University Düsseldorf, Düsseldorf, Germany; 13 Department of Endocrinology and Diabetology, University Hospital Düsseldorf, Heinrich Heine University, Düsseldorf, Germany; 14 Institute of Genetic Epidemiology, Helmholtz Zentrum München, Neuherberg, Germany; 15 Institute of Medical Informatics, Biometry and Epidemiology, Chair of Genetic Epidemiology, Ludwig-Maximilians-Universität, Munich, Germany; 16 Institute of Bioinformatics and Systems Biology, Helmholtz Zentrum München, Neuherberg, Germany; 17 Department of Physiology and Biophysics, Weill Cornell Medical College in Qatar, Qatar Foundation, Doha, Qatar; 18 Department of Mathematics, Technische Universität München, Garching, Germany; University of Melbourne, AUSTRALIA

## Abstract

Biological systems consist of multiple organizational levels all densely interacting with each other to ensure function and flexibility of the system. Simultaneous analysis of cross-sectional multi-omics data from large population studies is a powerful tool to comprehensively characterize the underlying molecular mechanisms on a physiological scale. In this study, we systematically analyzed the relationship between fasting serum metabolomics and whole blood transcriptomics data from 712 individuals of the German KORA F4 cohort. Correlation-based analysis identified 1,109 significant associations between 522 transcripts and 114 metabolites summarized in an integrated network, the ‘human blood metabolome-transcriptome interface’ (BMTI). Bidirectional causality analysis using Mendelian randomization did not yield any statistically significant causal associations between transcripts and metabolites. A knowledge-based interpretation and integration with a genome-scale human metabolic reconstruction revealed systematic signatures of signaling, transport and metabolic processes, i.e. metabolic reactions mainly belonging to lipid, energy and amino acid metabolism. Moreover, the construction of a network based on functional categories illustrated the cross-talk between the biological layers at a pathway level. Using a transcription factor binding site enrichment analysis, this pathway cross-talk was further confirmed at a regulatory level. Finally, we demonstrated how the constructed networks can be used to gain novel insights into molecular mechanisms associated to intermediate clinical traits. Overall, our results demonstrate the utility of a multi-omics integrative approach to understand the molecular mechanisms underlying both normal physiology and disease.

## Author Summary

Biological systems operate on multiple, intertwined organizational layers that can nowadays be accesses by high-throughput measurement methods, the so-called ‘omics’ technologies. A major aim in the field of systems biology is to understand the flow of biological information between the different layers at a systems level in both health and disease. To unravel the complex mechanisms underlying those molecular processes and to understand how the different functional levels interact with each other, an integrated analysis of multiple layers, i.e. a ‘multi-omics‘ approach is required. In our present study, we investigate the relationship between circulating metabolites in serum and whole-blood gene expression measured in the blood of individuals from a population-based cohort. To this end, we constructed a correlation network that displays which transcript and metabolite show the same trend of up- and down-regulation. We derived a functional characterization of the network by developing a novel computational analysis. The analysis revealed systematic signatures of signaling, transport and metabolic processes on both a regulatory and a pathway level. Moreover, integrating the network with associations to clinical markers such as HDL-cholesterol, LDL-cholesterol and TG identified coordinately activated pathways or modules which might help to assess the molecular machinery behind such an intermediate phenotype.

## Introduction

Blood is a connective tissue, which not only ensures nutrient and oxygen supply of all organs of the human body, but also the communication between them. Among the variety of key tasks performed by blood are immunological functions through white blood cells. Due to its diverse functionality, blood is heterogeneous and complex in its composition. Besides cellular constituents, which can be roughly divided into red and white blood cells, blood mainly consists of plasma. Plasma represents the aqueous phase containing proteins, peptides, signaling molecules and steroid hormones, but also other metabolites (e.g. carbohydrates, amino acids and lipids) which are consumed and released by the organs. This unique composition of blood, agglomerating both metabolic and transcriptional variation carrying molecular signatures of system-wide processes, together with its minimally invasive accessibility, makes blood a widely used system for integrative biomedical research [1,2].

With the development of high-throughput *omics* technologies for different levels of molecular organization, a systematic analysis of biological mechanisms underlying the functionality (or dysfunctionality) of a system became possible. In the case of transcriptomics data, an established framework to systematically investigate the constituents of involved biological processes and their interactions are network-based approaches, where pairwise associations between molecular entities (nodes) are modeled as network edges. Such studies commonly identify context-specific functional modules [3], but also global co-expression networks [4] from different organisms [5] and cell types [6]. When particularly focusing on the blood system, several studies investigated the co-regulation of transcripts either from single white blood cell types or whole blood samples. For example, regulatory networks [7,8] or global gene co-expression networks [9–11] were constructed from B- and T-cells to investigate pathways and mechanisms involved in the immune response. Further examples using whole blood data include the identification of disease related gene networks [12,13] or molecular signatures of distinct human vaccines captured in blood transcriptional modules [14].

Similarly, for metabolomics data a variety of studies extensively analyzed interactions between metabolites in various tissues, conditions and species [15–17]. Regarding blood measurements, we and others recently systematically characterized molecular interactions in the blood metabolome [18–21]. Utilizing Gaussian graphical models and serum metabolomics data from more than 1000 participants of a population cohort we were able to show that correlations between circulating blood metabolites resemble known metabolic pathways [22]. Furthermore, we have shown that these data-derived metabolic networks can be useful in a variety of applications, e.g. for the functional annotation of unknown metabolites [23] or to identify sex-specific serum metabolome differences [24].

The integration of multiple *omics* measurements (e.g. gene expression levels and metabolite concentrations) is an area of active research with many successful applications investigating the interplay between multiple organizational layers of a biological system [25–28]. However, only few studies with large sample sizes focused on a combined analysis in human blood. One recent example is the work of Inouye *et al*, who analyzed whole blood transcriptomics data in combination with blood lipid measurements and metabolites from a Finnish population cohort [29]. In their study, the authors associated a module of highly co-expressed genes with 134 blood metabolic markers in the context of heart disorders and identified a link between the immune system and circulating metabolites. The study by Inouye *et al* was among the first to provide clear evidence for this immune system link in blood, suggesting that gene expression in white blood cells is responsive to changing blood metabolite levels. Thus, it can be concluded that even if not cell-specific, the signals derived from whole blood data still reflect organism-wide processes. This is also in line with previous studies conducted on whole blood transcriptomics or metabolomics data separately [1,30,31].

**Fig 1:**
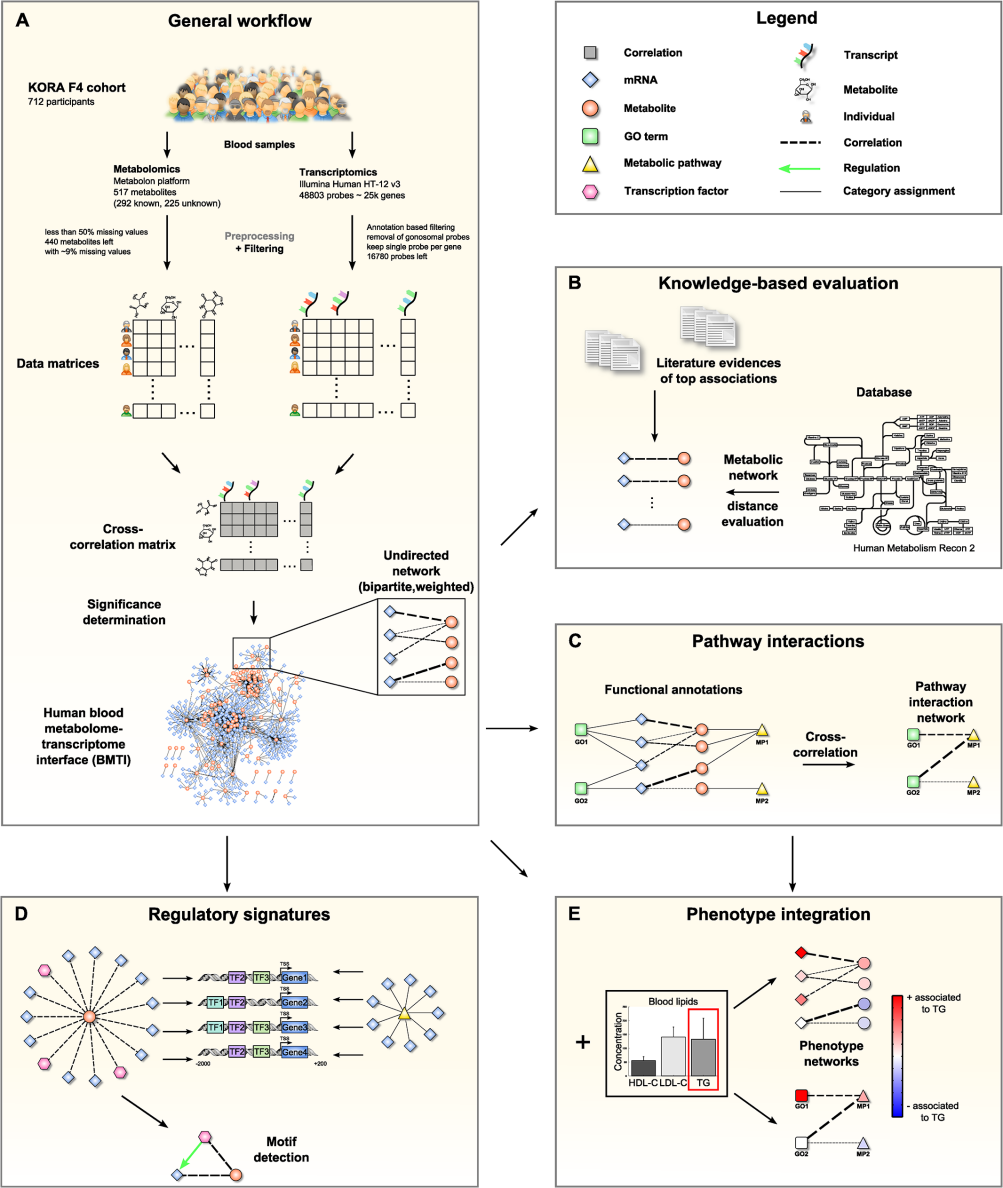
Data integration and network analysis workflow for the blood metabolome-transcriptome interface (BMTI). **A:** We analyzed fasting serum metabolomics and whole blood transcriptomics data from 712 samples of the KORA F4 cohort. After preprocessing and filtering, a cross-correlation matrix between 440 distinct metabolites and 16,780 unique, gene-mapped probes was calculated. The correlation matrix was transformed into a bipartite network by applying a statistical significance threshold. **B:** Scientific literature was screened for biological evidence for the strongest metabolite-mRNA associations. All correlating metabolite-mRNA pairs contained in the human metabolic model Recon2 were systematically evaluated with respect to their distance in the metabolic pathway network. **C:** Aggregated z-scores for each functional annotation were calculated. A pathway interaction network (PIN) was then constructed via cross-correlation of scores between pairs of functional annotations. **D:** For each metabolite contained in the BMTI, we investigated the promoter regions of associated transcripts for shared regulatory signatures. Similarly, shared regulatory signatures within and between metabolic pathways were examined. As a final step, we identified specific regulatory motifs in the BMTI. **E:** Both BMTI and the PIN were integrated with the results from an association analysis to the three intermediate physiological traits (HDL-C, LDL-C and TG).

The aim of the present study was to make use of the joint power of metabolomic and transcriptomic profiling to comprehensively characterize the complex interplay between serum metabolomics and whole-blood transcriptomics data. While serum metabolomics represent a footprint of whole-body processes, blood transcriptomics data will mainly reflect immune system processes through white blood cells. To this end, we analyzed metabolomics and transcriptomics measurements of 712 individuals from the German population study KORA (“Kooperative Gesundheitsforschung in der Region Augsburg”), comprising 440 metabolites and 16,780 genes after filtering. We constructed a global correlation network to elucidate the complex interplay and regulation between these *omics* layers (Fig 1A). The correlation analysis takes advantage of the naturally occurring variation from individual to individual, which we assume to carry a systematic footprint of the coregulation of metabolites and mRNAs. Such an integrative approach was recently termed “systems genetics”, providing a global view on the information flow between the various biological scales [32]. We deliberately left out an analysis of metabolite-metabolite and transcript-transcript correlations, which were rigorously investigated in the above-mentioned earlier studies. Instead, we specifically sought to assess the interconnection and information flow between the two *omics* layers.

The manuscript is organized as follows: In the first part, we systematically characterize the *blood metabolome-transcriptome interface* (BMTI) using different strategies. First, we manually investigated the strongest associations and provide evidence from literature wherever possible. Moreover, using a Mendelian randomization (MR) approach, we examined potential causal relationships between metabolites and transcripts. Second, using the most recent genome-wide human metabolic network *Recon 2* [33], we systematically analyzed correlations between metabolites and transcripts at a pathway level (Fig 1B). Third, we developed a novel network clustering approach based on functional annotations, leading to a *pathway interaction network* (PIN) that allows for fast functional interpretation of the BMTI and furthermore provides insights into the cross-talk among distinct molecular pathways (Fig 1C). In the second part of this manuscript, we demonstrate how the identified networks can be used as a resource to further investigate the link between metabolism and gene regulation by two different applications. First, we investigated whether a common regulatory signature is observable from transcripts connected to the same metabolite or to metabolites that are part of the same metabolic pathways. For this purpose, we analyzed promoter regions of the genes for overrepresented transcription factor binding sites (Fig 1D). Second, we integrated the metabolome-transcriptome and the pathway interaction network with associations to high density lipoprotein cholesterol (HDL-C), low density lipoprotein cholesterol (LDL-C) and triglycerides (TG), which are well-known risk factors for cardiovascular disease [34]. To this end, we mapped the results of linear regressions between these clinical lipid parameters with metabolites and mRNAs onto the networks (Fig 1E). Finally, we demonstrate the potential of our systems genetics approach to generate novel hypothesis by combining results from all separate analysis steps and establish an association between the branched-chain amino acid pathway and the levels of plasma TG and HDL-C.

All network results are available to the scientific community as interactive versions in graphml and Cytoscape format (S1 Dataset).

## Results

### The human blood metabolome-transcriptome interface

For this study, we focused on a subset from the KORA F4 cohort with simultaneously available metabolomics and transcriptomics data. After quality control and filtering, the data set comprised of 712 human blood samples (354 males, 358 females) with gene expression data of 16,780 uniquely mapping gene probes and metabolite concentrations of 440 metabolites (Fig 1A, see Materials and Methods for details). 186 of these 440 metabolites were not chemically identified, which is marked by a metabolite name starting with “X-”throughout this manuscript. Both gene expressions and metabolite concentrations were log transformed and adjusted for age and sex effects. Pairwise Spearman’s rank correlations between the measured mRNAs and metabolites were then calculated. We used this correlation method to account for possible non-linear associations and to ensure robustness against outliers. Note that for this particular dataset, Spearman and Pearson correlations produced almost identical results (S1 Fig). S1 Table provides a full list of identified significant associations between blood metabolites and transcripts.

**Fig 2:**
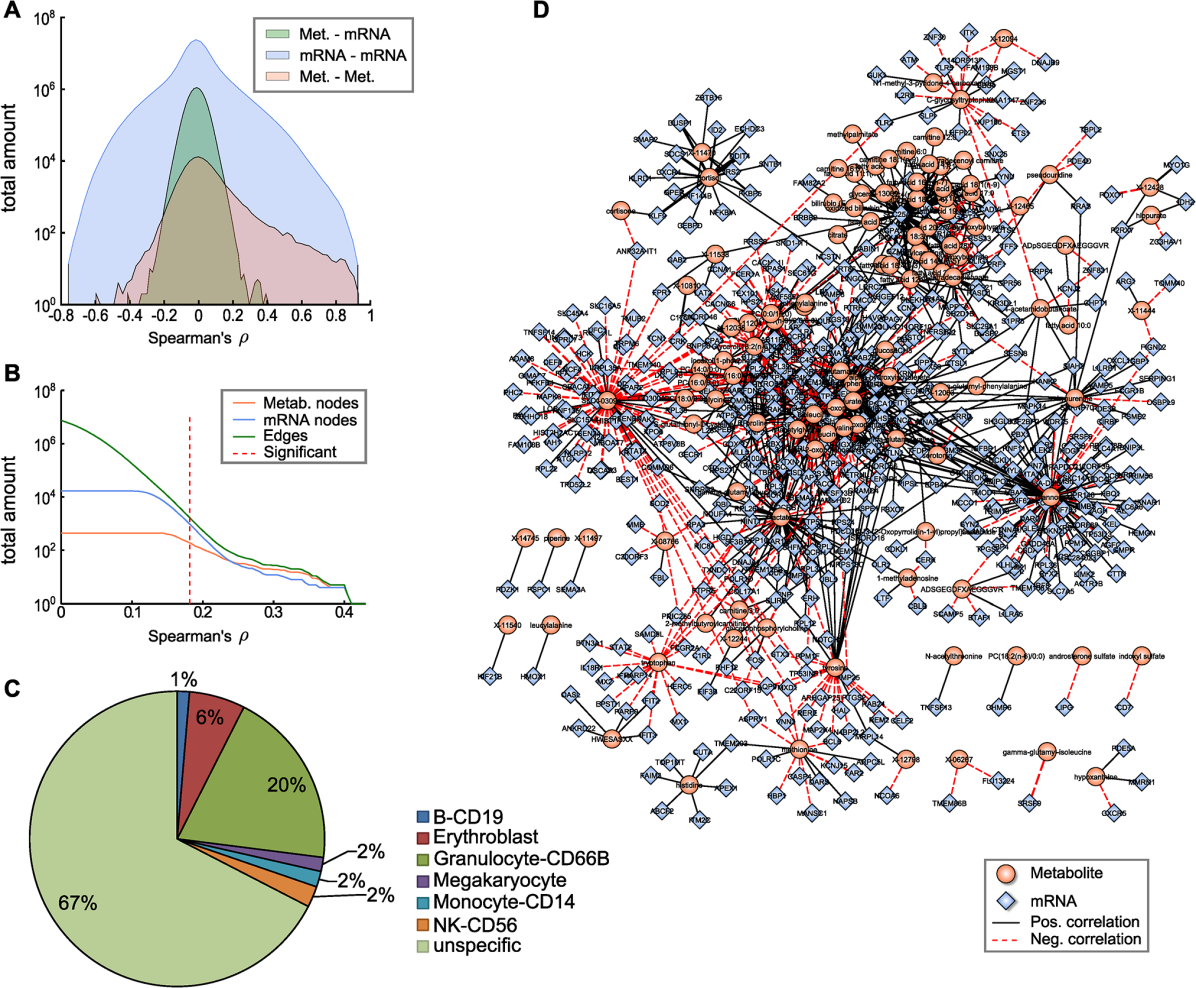
The blood metabolome-transcriptome interface. **A:** Distributions of correlation coefficients for metabolite-mRNA, mRNA-mRNA and metabolite-metabolite associations. **B:** Number of nodes and edges as a function of the absolute correlation coefficient. Red dotted line represents the correlation cutoff used in this study (0.01 FDR). **C:** Percentages of blood cell type specific markers contained in the BMTI. **D:** Visualization of the blood metabolome-transcriptome interface. The correlation network consists of 114 metabolites and 522 transcripts connected by 1109 edges. Edge widths represent correlation strengths.

Metabolite-mRNA Spearman correlation coefficients were symmetrically distributed around zero (mean:−4.5 × 10^−4^ ± 0.0433, Fig 2A) with a maximum absolute correlation value of *ρ* = 0.56. Moreover, the distribution of inter-omics correlations showed a rather narrow shape, indicating generally lower correlations when compared to the intra-omics correlations (mRNA-mRNA, metabolite-metabolite). The metabolite-metabolite distribution was strongly skewed for positive correlation values, which is in accordance with our previous findings on a different metabolomics panel [22]. In contrast, the mRNA-mRNA distribution displayed a broad and symmetric distribution of correlation values (Fig 2A).

We then generated a weighted bipartite network of metabolites and transcripts by constructing an edge between a pair of metabolite and transcript if the respective correlation was significant with a false discovery rate (FDR) of 0.01. This corresponded to an absolute correlation cutoff of ~0.181 and a p-value threshold at 1.07 × 10^−6^. Obviously, the number of edges in a correlation network heavily depends on the chosen threshold. It has been shown in previous studies that a biologically reasonable threshold can be found by investigating network density as a function of the correlation cutoff value [35]. According to that study, a cutoff value slightly above the minimal density combined with a decreasing number of nodes and edges leads to biologically meaningful results. As indicated in Fig 2B, a clear decline in the number of included nodes and edges can be observed for increasing correlation threshold levels beginning between correlation values of 0.15 and 0.25. Minimal network density was reached for a correlation threshold value between 0.13 and 0.18 (S2 Fig). Notably, applying the above-mentioned conventional statistical significance threshold to our data set precisely coincides with the network density-based threshold described by [35].

The resulting network, subsequently called the blood metabolome-transcriptome interface (BMTI), consisted of 636 nodes (114 metabolites, 522 transcripts) and 1109 edges, corresponding to a total network connectivity of ~0.015% (Fig 2B and 2D). Out of the total number of edges, 63% (699) were positive correlations and 37% (410) were negative correlations. The metabolite showing the highest degree was mannose, with significant correlations to 98 different transcripts. In contrast, the mRNA with the highest connectivity was *SLC25A20* with 37 metabolites attached (S1 Table).

We used data from the DILGOM study, which included NMR metabolomics as well as transcriptomics data for 518 individuals, for independent replication of our correlations. In total, 17 metabolites (11 amino acids, 3 lipids, 2 carbohydrates and 1 belonging to the energy metabolism) overlapped between the KORA F4 dataset and the DILGOM study, which allowed us to investigate the replication of 211 edges (~19% of the BMTI). 61 out of the 211 edges (~29%) reached a nominal significance (p-value < 0.05) in the DILGOM study of which 38 (~18%) remained significant after multiple testing correction (FDR < 0.05, see S2 Table).

To investigate the possible origins of the metabolite-transcript correlations, we compared all genes represented in the BMTI with 1) two *a priori* defined blood cell type-specific marker gene lists, and 2) a database of more general tissue gene expression signatures (see Materials and Methods). For the first part, we used a list of genes derived from Palmer et al. [36] comprising 907 specifically expressed genes for 5 different blood cell types (leukocytes only) and a second list derived from the HaemAtlas as generated by Watkins et al. [37] comprising 1,716 genes characterizing 9 different blood cell types. For the second part, we used the HECS database from Shoemaker et al. [38] containing information for more than 6,000 genes and 84 tissues. Both comparisons in 1) showed that most of the BMTI genes (85% and 67%, respectively) were not specifically attributable to any blood cell type (see Fig 2C and S3 Table). The remaining genes could be assigned to the respective measured cell types, with granulocytes making up the largest blood cell faction in both cases (8% and 20%, respectively) and only minor signals for the other blood cell types. A similar result was observed when comparing the BMTI genes to the HECS database. 52% of the BMTI genes showed no tissue specificity, while 12 out of the 15 strongest tissue signatures where either blood cells or blood related tissues (S3 Table).

### Strong network edges in the BMTI represent known pathway mechanisms

As a first step to characterizing the BMTI, we performed a manual literature lookup for the strongest absolute correlations in the network (Fig 1B). In the following, we provide a detailed discussion of the 25 strongest edges (Table 1). Notably, most of the top 25 identified associations reflect biochemically reasonable interactions like transport processes of lipids, but also regulatory signatures between signaling metabolites and transcription factors.

**Table 1:** Top 25 network edges.

Metabolite	mRNA	Spearman's ρ	p-value
cortisol	DDIT4	0.56	7.71E-59
1-oleoylglycerol (1-monoolein) | Glycerol(18:1(n-9)/0:0/0:0)	HDC	-0.41	2.01E-29
oleate181n9 | oleate (18:1n9)	SLC25A20	0.40	2.30E-29
palmitate (16:0) | fatty acid 16:0	SLC25A20	0.40	3.96E-29
1-oleoylglycerol (1-monoolein) | Glycerol(18:1(n-9)/0:0/0:0)	SLC45A3	-0.40	3.59E-28
dihomolinoleate202n6 | dihomo-linoleate (20:2n6)	SLC25A20	0.38	8.63E-26
linoleate (18:2n6) | fatty acid 18:2(n-6)	SLC25A20	0.37	1.55E-24
stearate (18:0) | fatty acid 18:0	SLC25A20	0.37	2.02E-24
eicosenoate (20:1n9 or 11) | fatty acid 20:1(n-9/n-11)	SLC25A20	0.37	2.67E-24
10-nonadecenoate (19:1n9) | fatty acid 19:1(n-9)	SLC25A20	0.36	9.07E-24
cortisol	SOCS1	0.36	2.19E-23
3-hydroxybutyrate (BHBA)	SLC25A20	0.35	1.77E-22
5,8-tetradecadienoate	SLC25A20	0.35	3.85E-22
palmitoleate (16:1n7) | fatty acid 16:1(n-7)	SLC25A20	0.35	4.60E-22
10-heptadecenoate (17:1n7) | fatty acid 17:1(n-7)	SLC25A20	0.35	7.87E-22
1-oleoylglycerol (1-monoolein) | Glycerol(18:1(n-9)/0:0/0:0)	GATA2	-0.35	2.63E-21
cortisol	KLF9	0.34	5.21E-21
linolenate [alpha or gamma; (18:3n3 or 6)] | fatty acid 18:3(n-3/n-6)	SLC25A20	0.34	5.13E-21
cortisol	DUSP1	0.34	7.59E-21
margarate (17:0) | fatty acid 17:0	SLC25A20	0.34	3.50E-20
myristate (14:0) | fatty acid 14:0	SLC25A20	0.32	8.05E-19
1-oleoylglycerol (1-monoolein) | Glycerol(18:1(n-9)/0:0/0:0)	C1ORF186	-0.32	4.72E-18
5-dodecenoate (12:1n7) | fatty acid 12:0(n-7)	SLC25A20	0.32	3.74E-18
isoleucine	ABCG1	-0.32	3.39E-18

We observed particularly strong effects for lipid metabolism, especially around the mitochondrial transporter SLC25A20. Pipe symbols (|) separate alternative metabolite names.

The strongest association in the dataset was observed between cortisol and *DNA-Damage-Inducible Transcript 4* (*DDIT4*, *ρ* = 0.55, p-value = 7.70 × 10^−59^), which are known to play a role in stress response [39]. Cortisol is a glucocorticoid whose release is mainly induced by exogenous stress. Via binding to the glucocorticoid nuclear receptor (GR, official gene symbol *NR3C1*), it regulates various cellular processes like carbohydrate metabolism and the immune system by direct activation of target genes [40]. Remarkably, *DDIT4* was identified as a GR target gene in mouse hepatocytes [41], rat hippocampus [42] and also in human peripheral blood lymphocytes [43] delivering a potential explanation of an indirect association for the observed correlation. Another GR target gene associated to cortisol is *Suppressor Of Cytokine Signaling 1* (*SOCS1*, *ρ* = 0.36, p-value = 2.19 × 10^−23^), a major constituent of the cytokine signaling pathway and inflammatory response [44]. We observed further top 25 correlations involving cortisol for *Kruppel-Like Factor 9* (*KLF9*) and *Dual Specificity Phosphatase 1* (*DUSP1*) (*ρ* = 0.34, p-value = 5.20 × 10^−21^; *ρ* = 0.34, p-value = 7.58 × 10^−21^, respectively). *KLF9* is a ubiquitously expressed transcription factor involved in the regulation of diverse biological processes like cell development and differentiation in adipogenesis [45]. *DUSP1* is an enzyme involved in the response to environmental stress [46]. Interestingly, for both transcripts, a cortisol-dependent regulation was already observed in epidermal cells [47] and peripheral blood mononuclear cells [48].

Another metabolite showing several strong associations to blood transcripts was 1-monoolein, which belongs to the class of monoacylgylcerols. This particular class of metabolites are bioactive compounds recently identified to be involved in various signaling processes of the immune system [49,50]. The source of 1-monolein in humans is not fully understood. Experiments in rodents suggest that dietary 1,3-diacylglycerols are preferentially digested to 1-monoacylglycerols and free fatty acid in the small intestine, making dietary 1,3-diacylglycerols containing an oleoyl moiety at position sn-1 or sn-3 a plausible source of 1-monoolein [51]. In our analysis, 1-monoolein showed a strong negative correlation to four transcripts – *GATA Binding Protein 2* (*GATA2*), *Histidine Decarboxylase* (*HDC*), *Solute Carrier Family 45*, *Member 3* (*SLC45A3*) and *Chromosome 1 Open Reading Frame 186* (*C1ORF186*) (*ρ* between -0.41 and -0.32, p-values between 2.01 × 10^−29^ and 4.72 × 10^−18^). *HDC* is a cytosolic enzyme that catalyzes the conversion of histidine to histamine and thus represents an important immune system trigger molecule [52]. In addition, *GATA2*, a key regulator of gene expression in hematopoietic cells [53], *C1ORF186* and *SLC45A3*, two membrane-bound proteins, were all identified to play a role in the immune response [13].

Carnitine-acylcarnitine translocase (*SLC25A20*) occurred in 15 of the 25 top ranked correlations. This gene encodes an enzyme which transports acylcarnitines, i.e. the transport variant of fatty acids, into the mitochondria for subsequent ß-oxidation. Interestingly, the majority of *SLC25A20*-associated metabolites among our top 25 correlations belonged to the class of long chain fatty acids (11 long chain fatty acids, 2 essential fatty acids, 1 medium chain fatty acid, 1 ketone body), which is in accordance with its function as a lipid transporter. Of note, among the metabolites associated with *SLC25A20* beyond the top 25 correlations were also 5 acylcarnitines, although at lower correlation values.

We observed a significant, negative correlation between isoleucine and *ATP-Binding Cassette Sub-Family G Member 1* (*ABCG1*, *ρ* = −0.32, p-value = 3.39 × 10^−18^). It has been shown previously that circulating levels of branched-chain amino acids (BCAAs) affect a variety of metabolic processes such as glucose and lipid metabolism [54]. ABCG1 is a major player of lipid metabolism, controlling the transfer of cholesterol from peripheral macrophages to exogenous HDL [55]. Interestingly, an association between circulating BCAA levels and plasma HDL-C levels was also observed in a recent population study [56] and in a previous paper on the same population cohort used in the present study [57].

### Causal analysis of BMTI edges

To assess whether metabolite-transcript links in BMTI contain causal effects, we performed a Mendelian randomization analysis [58]. For each metabolite-mRNA edge, we tested both the causal directions metabolite→mRNA and mRNA→metabolite given that adequate instrumental variables were available. As instruments we used SNP lists from previously published GWAS studies. After filtering for strong instrumental variables, we were left with 15 SNPs identified by a metabolomics GWAS study [23] associated to 16 metabolites in the BMTI. Moreover, for 157 mRNAs in the network, we selected 192 SNPs from [59]. In total, we tested the causal relationship of 440 BMTI edges (~40%) of which 60 could be tested bi-directional. In the BMTI, 42 metabolite-mRNA pairs (19 mRNA→metabolite; 23 metabolite→mRNA) showed a nominally significant (p-value < 0.05) causal effect. At an FDR of 0.05, none of the tested pairs remained significant (S4 Table).

### Model-based evaluation reveals systematic signatures of metabolic reactions

**Fig 3:**
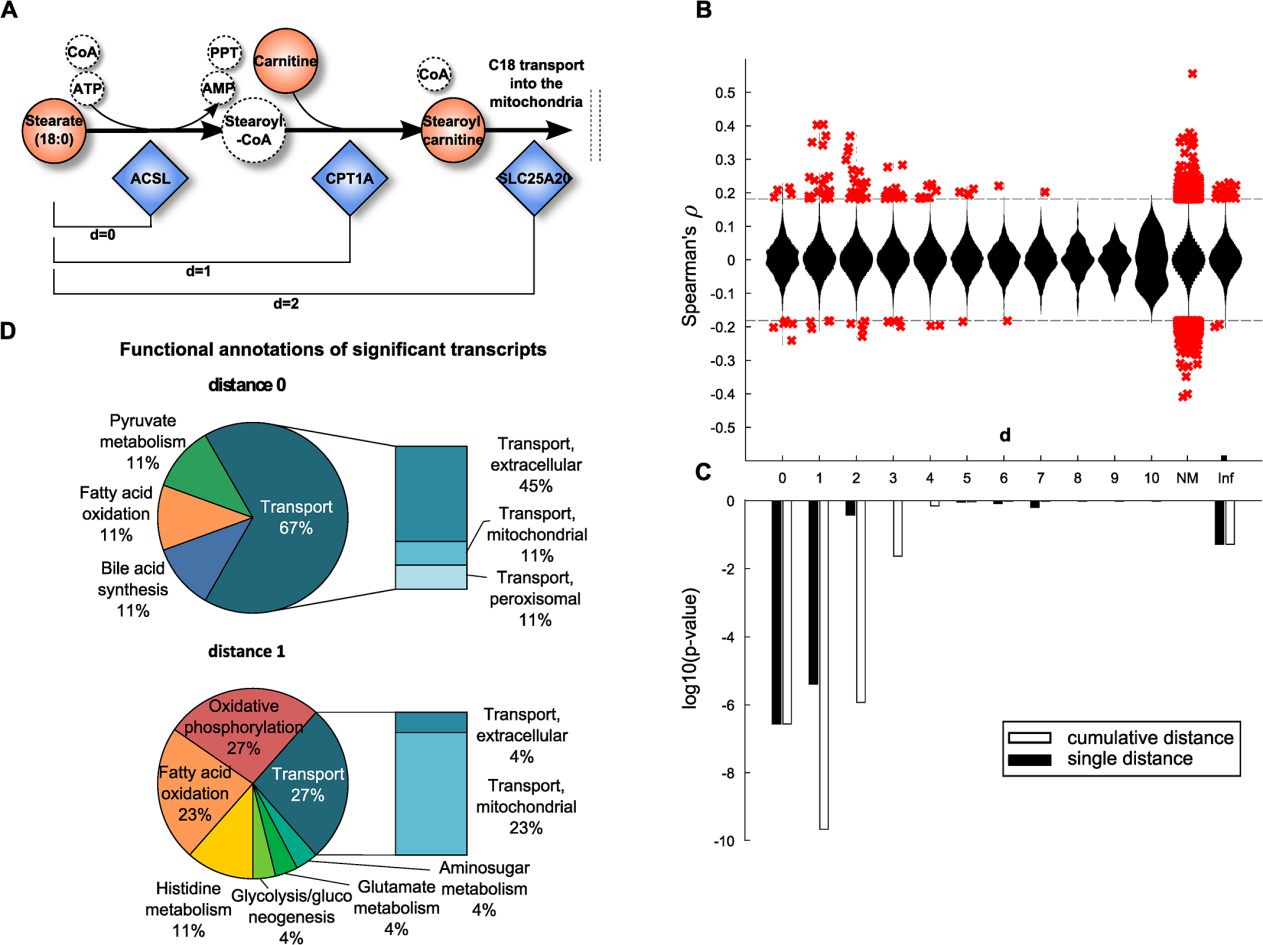
Model-based evaluation of metabolite-mRNA correlations. **A:** Schematic representation of the mitochondrial carnitine-shuttle with an explanation of network distance calculation. Note that co-factors are only illustrated for completeness, but are not considered for the calculation of the shortest path between two compounds. Dashed circles indicate unmeasured metabolites. **B:** Spearman correlation coefficient plotted against the number of pathway steps in human Recon 2. Significant correlations, i.e. those present in the BMTI are displayed as red crosses, whereas all non-significant correlations are plotted as a distribution. NM: no mapping. A distance of infinity (Inf) was assigned if there was no connection in Recon 2. **C:** Enrichment of significant correlations as determined by Fisher’s exact test. Black bars indicate log10 p-values assessing whether we observe more significant correlations for that particular distance than expected by chance. White bars represent the same test, only for a cumulative distance (i.e. “up to a distance of x”). **D:** Functional annotations of significantly associated transcripts at distances 0 and 1. At both distances, mainly transcripts belonging to the transport, energy, lipid and amino acid subsystems were observable.

In order to further reveal the underlying mechanisms determining the observed associations, we systematically analyzed whether correlating pairs of metabolites and transcripts (i.e. enzymes) correspond to the structure of the underlying metabolic network. Specifically, we investigated if strong metabolite-transcript edges of the BMTI tend to be in close proximity within biochemical pathways. All pairwise associations between metabolites and transcripts were mapped to their corresponding network nodes in the Human Recon 2 metabolic network reconstruction [33]. As a measure for metabolic network proximity, the length of the shortest path connecting each metabolite-enzyme pair was determined (Fig 3A). This measure is based on the common assumption that the shortest connection between two network entities corresponds to the biologically reasonable one [22,60]. To avoid potential biologically meaningless shortcuts, we removed co-factors and currency metabolites prior to the analysis (see Methods section for details and S5 Table for a list of removed metabolites).

We could map 121 metabolites and 1,467 enzymes out of the 254 metabolites with known identity and 16,780 transcripts onto the metabolic network, respectively. While most pairwise correlation coefficients were closely distributed around zero for all investigated network distances, a distinct pattern was observable for statistically significant correlations. The majority of significantly correlating pairs accumulated at short distances and was dominated by positive correlations (Fig 3B). To determine the significance of this observation, one-tailed Fisher’s exact tests were performed by either considering each distance individually or by aggregating all pairs up to a particular distance. The latter aggregation analysis combines all transcript-metabolite pairs which are reachable up to a certain number of steps (biochemical reactions) in the metabolic network. For both cases, we observed a substantial overrepresentation of significantly correlating pairs at short distances (Fig 3C). The strongest signals are observed for pairs that take part either directly in the same reaction (d = 0) or for those which are just one reaction apart (d = 1). For the cumulative distances we also observed significant enrichment up to a distance of d = 2 reactions. Proportions of significant and non-significant pairs per distance are given in S3 Fig and a detailed view on an exemplary path of length 2 is depicted in S4 Fig

To further characterize the underlying biochemical pathways, we calculated frequencies of functional annotations from Recon 2 among the significant associations for pathway distances 0 to 2 (Fig 3D and S5 Fig). At a distance of 0, we identified mainly transport reactions (67%) accompanied by reactions belonging to lipid metabolism (bile acid synthesis 11%, fatty acid oxidation 11%) and carbohydrate metabolism (pyruvate metabolism 11%). The transport reactions can be further subdivided into extracellular transport (45%), or mitochondrial transport (11%) and peroxisomal transport (11%). Similar signals can be found at distances of 1 and 2, where we additionally identified reactions belonging to energy metabolism (oxidative phosphorylation 27%) and amino acid metabolism (histidine metabolism 11%, glutamate metabolism 4%).

Taken together, the BMTI captured a systematic signal of metabolite-enzyme associations to be in close proximity when mapped onto a global metabolic network. Moreover, the strongest signals found for pathway distances of 0, 1 or 2 reflect distinct metabolic reactions mainly belonging to lipid, energy and amino acid metabolism, and transport mechanisms.

### Functional annotation-based aggregation of the BMTI reveals cross-talk between pathways

Up to this point, our analysis was a reaction-centered approach limited to single edges only, thereby neglecting the global network structure and cross-talk between pathways captured in the BMTI. To derive a comprehensive functional description of the biological modules included in the BMTI, we developed a novel approach based on functional annotations which provides an integrated view on cellular processes. Briefly, the approach consists of three steps: First, we used pathway annotations to define groups of functionally related metabolites and transcripts. For metabolites, we used metabolic pathway annotations provided with the metabolomics dataset, and for transcripts we downloaded the Gene Ontology (GO) slim annotations. Second, an aggregated z-score (aggZ-score) was calculated for each functional category. Third, we calculated correlations between aggZ-scores of all functional categories. A schematic overview of this multi-step approach is provided in S6 Fig and described in more detail in the Material and Methods. A full list of the resulting categorical correlations can be found in S6 Table.

**Fig 4:**
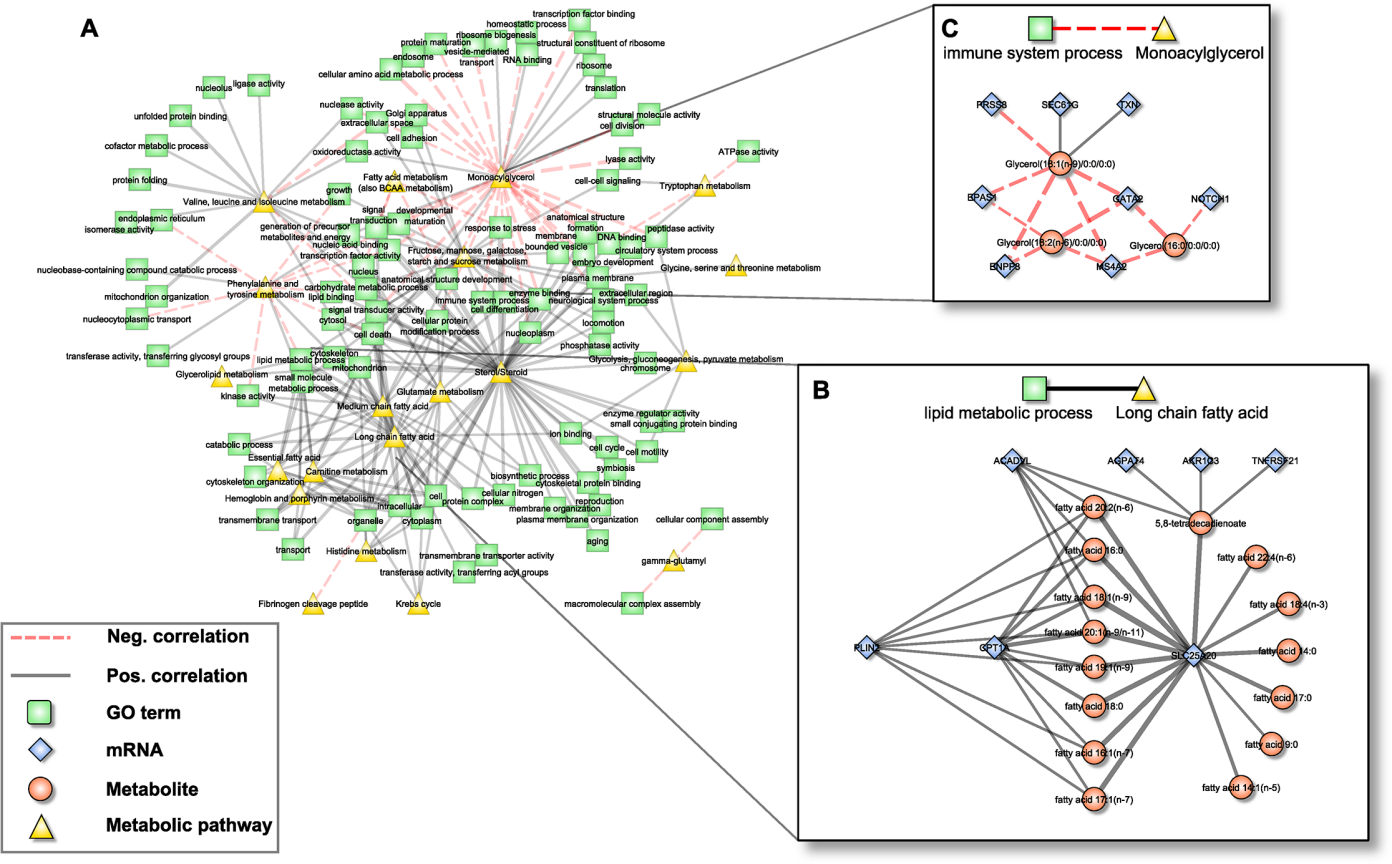
Pathway interaction network (PIN). **A:** Bipartite correlation network, where each node represents either a metabolic pathway or a gene set summarized in a GO term, while edges between them represent the correlation of the respective aggZ-scores. **B+C:** Expanded view on exemplary pathway interactions. Note that zoomed parts correspond to subnetworks from Fig 2D that are explained by one single link of the PIN. Edge widths represent correlation strengths.

We again constructed a network (the pathway interaction network, PIN) by drawing edges between significantly correlated categories. Interestingly, even when applying a stringent Bonferroni-corrected threshold (*α* = 0.01, p-value ≤ 2.2 × 10^−6^) this resulted in an overly dense connected network of 166 nodes and 1220 edges. To generate a visually interpretable version of this network, an ad-hoc stringent threshold of p-value ≤ 1.0 × 10^−11^ was applied to draw the network. This resulted in a PIN consisting of 113 nodes (93 GO terms, 20 metabolic pathways) connected by 244 edges (196 positive correlations, 48 negative; Fig 4A). Remarkably, we observed a high conformity between linked metabolic pathways and gene annotations. For example, the metabolic pathway “carnitine metabolism” was connected to the biological processes “lipid metabolic process” and “transmembrane transport”. Moreover, it was linked to the cellular component “mitochondrion”, indicating transport processes of fatty acids into the mitochondrion for subsequent ß-oxidation. Further biologically reasonable pairs were “Valine, Leucine and Isoleucine metabolism” and “Glutamate metabolism” attached to “cellular nitrogen compound metabolic process”. As a last example, “Steroid/Sterol” was connected to “response to stress” and “signal transducer activity”, pointing to an interaction between hormones and regulation of gene expression. In the following, we examine two selected category-category relationships in detail, including the individual metabolites and gene transcripts that gave rise to the association.

#### Scenario one: Fatty acid metabolism

The first scenario contained the metabolic pathway “long chain fatty acid” and the gene ontology annotation “lipid metabolic process” (Fig 4B). The subnetwork that induced this association consisted of 22 individual constituents (7 mRNAs, 15 metabolites) connected by 38 edges. Notably, this subnetwork coincides well with the above-mentioned *fatty acid carnitine-shuttle*, i.e. the transport of long chain fatty acids into the mitochondrion for subsequent degradation. Within this subnetwork, 8 long chain fatty acids were jointly associated to *CPT1A* and *SLC25A20*, while 7 additional fatty acids were associated to *SLC25A20* alone. Moreover, *acyl-CoA dehydrogenase very long-chain* (*ACADVL*) and *Perilipin 2* (*PLIN2*), both involved in ß-oxidation and long chain fatty acid transport, were associated to 5 and 7 out of the 15 long chain fatty acids, respectively. Three further transcripts, *Tumor Necrosis Factor Receptor Superfamily*, *Member 21* (*TNFRSF21*), *Aldo-Keto Reductase Family 1*, *Member C3* (*AKR1C3*) and *1-Acylglycerol-3-Phosphate O-Acyltransferase 4* (*AGPAT4*) were correlated with 5,8-tetradecadienoate, a side product of oleate ß-oxidation. While *AKR1C3* and *AGPAT4* are enzymes mainly related to arachidonic acid metabolism and phospholipid metabolism, potentially indicating a branching point to other pathways of the lipid metabolism, *TNFRSF21* is involved in T-cell activation and immune regulation [61].

#### Scenario two: Monoacylglycerols and immune-related transcripts

Since whole blood transcriptomics measurements should mainly reflect the immune system, we have chosen the association between the metabolic class of “monoacylglycerols” and the GO-term “immune system process” as a second scenario (Fig 4C). The corresponding subnetwork contained 11 nodes (3 metabolites, 8 transcripts) and 14 edges (2 positive, 12 negative). The monoacylglycerols included 1-oleoylglycerol, 1-linoleoylglycerol and 1-palmitoylglycerol, which only differ in the attached fatty acid residues. It has been shown that monoacylglycerols are not merely intermediate lipid substances, but may also act as signaling molecules. For example, 2-arachidonoylglycerol is a known ligand of cannabinoid receptors, which are involved in the regulation of several biological processes including inhibition of pro-inflammatory and other immune system related processes [62,63]. Among the genes summarized by the term “immune system process”, *GATA2*, *Endothelial PAS Domain Protein 1* (*EPAS1*) and *Notch1* are key regulators of hematopoiesis and as such are involved in the differentiation process of immune cells [53,64,65]. Moreover, *EPAS1* and *thioredoxin* (*TXN*) are associated to the response to oxidative stress [64], whereas *Membrane-Spanning 4-Domains*, *Subfamily A*, *Member 2* (*MS4A2*) and *Ectonucleotide Pyrophosphatase/Phosphodiesterase 3* (*ENPP3*) are involved in allergic responses mediated via the IgE receptor [66]. The two remaining genes, SEC61G and PRSS8, are involved in the immune processes of antigen presentation and inflammation [67].

### Regulatory signatures captured by the integrated network

The BMTI contains a prominent “flower-like” network topology, i.e. many transcripts associated to a single metabolite. We therefore asked whether these coordinated changes around a metabolite and also the network topology can be explained by common transcriptional regulatory processes through transcription factors (TFs, Fig 1D). For the following analysis, we only considered metabolites linked to at least 3 transcripts. We analyzed the promoter regions of all connected genes for an enrichment of known transcription factor binding sites (TFBS) derived from the Jaspar database [68]. This resulted in significantly enriched transcription factor binding motifs for 46 single metabolites, 24 *subpathways* and 7 *superpathways*. The Methods section provides a detailed explanation of the process. A summary of all enriched TFBS can be found in S7 Table.

In total, out of the 205 binding motif matrices used in the analysis, 189 reached a significant enrichment in at least one of the metabolite-derived gene sets, indicating a generally prevalent common regulation. Across all lists of enriched TFBS identified from our network, the motifs that occurred most frequently were *Sterol Regulatory Element Binding Transcription Factor 2* (*SREBF2*), *Peroxisome Proliferator-Activated Receptor Gamma* (*PPARG;* Jaspar motifs *PPARG* and *PPARG*::*RXRA*) and *Nuclear Factor*, *Interleukin 3 Regulated* (*NFIL3*). *SREBF2* is a major regulator of cholesterol metabolism [69] while *PPARG* is known to be activated by fatty acid ligands, thereby regulating fatty acid ß-oxidation and other processes [70]. NFIL3 is a regulator specifically found in activated T cells, natural killer (NK) cells, and mast cells, involved in the regulation of the immune response and the circadian rhythm [71].

Branched-chain amino acids were among the metabolites most strongly connected to SREBF2 targets. Specifically, the transcripts correlating with isoleucine and valine show high enrichment of SREBF2 binding sites (p-value = 5.83 × 10^−8^ and p-value = 2.36 × 10^−10^, respectively; S7 Table). Moreover, considering all 172 genes associated to at least one metabolite from the entire branched-chain amino acid pathway (“Valine, leucine and isoleucine metabolism”) yielded significantly enriched binding sites for *SREBF1* and *SREBF2* (p-values 6.78 × 10^−10^ and 9.11 × 10^−10^, respectively; S7 Table). Both SREBs are important regulators in lipid homeostasis, including cholesterol and fatty acid biosynthesis, further indicating a regulatory cross-link between HDL-C, TG and BCAA metabolism.

**Fig 5:**
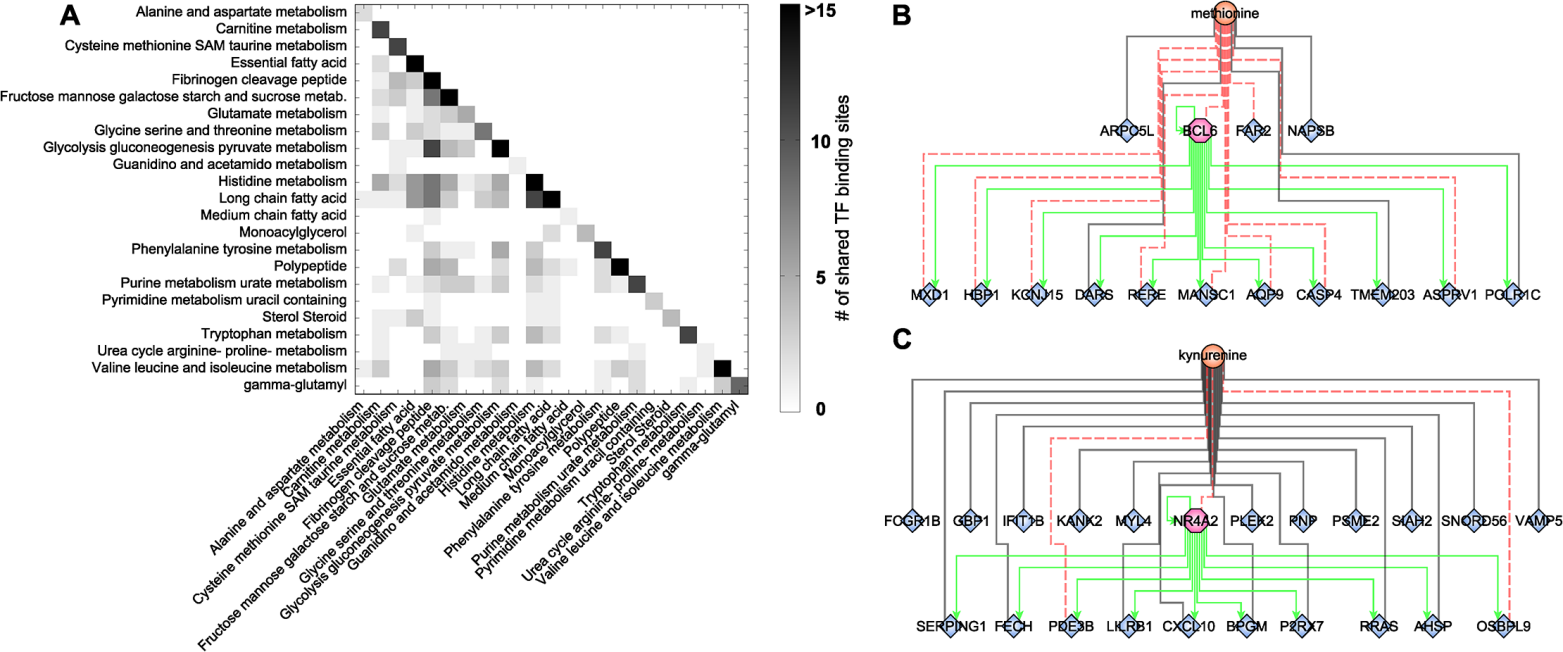
Transcription factor binding site analysis. **A:** Heatmap of shared significantly overrepresented TFBS between metabolic *subpathways*. Upper right triangle matrix was left out. Darker colors indicate a higher number of shared TF binding sites. **B+C:** Identified network motifs of TFs and their respective target genes associated to the same metabolite. Green arrows indicate regulation. Black lines indicate positive correlation; red dotted lines indicate negative correlation.

The highly interlinked network topologies of both the blood metabolome-transcriptome interface and the pathway interaction network suggest a strong coregulation between the different metabolites, processes, and pathways. As a second step of coregulation analysis, we inferred the number of pairwise shared significant TFBS to determine the extent of coregulation between single metabolites and metabolic pathways (S8 Table). At the single metabolite level, we found a maximum number of 27 shared TFs between histidine and X-03094 (S7 Fig). Moreover, this highly connected unknown metabolite shared 14 TFs with another unknown metabolite (X-12442) and with a peptide (HWESASXX). For the metabolic *subpathways*, we observed an overlap between “histidine metabolism” and the group of “long chain fatty acids” and between “glycolysis, gluconeogenesis, and pyruvate metabolism” and the group of “fibrinogen cleavage peptides” (11 shared TFs each; Fig 5A). On the level of *superpathways*, the highest number of shared TFBS was 4, identified between “carbohydrate” and “peptide metabolism” (S8 Fig). Overall, we found that TF binding sites are shared to a large extent, indicating a complex coregulation not only within but also between different processes and pathways.

To gain further insight into this coregulation, we determined transcription factors which also occur as transcripts in the BMTI. 165 out of the 189 transcription factors with available binding motif were contained in the filtered data set. Only 12 of these transcription factors displayed a significant correlation to any metabolite and are thus included in the BMTI. This observation is not completely unexpected given that TFs are regulated to a large extend at a post-transcriptional level [72]. Interestingly, for two out of these 12 TFs, we also observe enriched binding sites in the promoter region of the other genes connected to the same metabolite, i.e. a “triad” network motif consisting of a metabolite, a transcription factor and its target genes (Fig 1D, S7 Table).

The first transcription factor is *B-cell CLL/Lymphoma 6* (*BCL6*), a transcriptional repressor involved in the STAT-dependent interleukin 4 response of B-cells [73]. *BCL6* is negatively correlated with methionine and tyrosine in our network (Fig 5B). The TFBS enrichment analysis using all 15 genes connected to methionine within the BMTI resulted in a significant overrepresentation of the BCL6 binding motif (p-value = 5.71 × 10^−09^, 82% of the 15 promoter sequences contained at least one occurrence of the motif), while no significant enrichment was observable for the genes connected to tyrosine. The second motif was identified around *Nuclear Receptor Subfamily 4*, *Group A*, *Member 2* (*NR4A2*), which was associated to 7 metabolites in our network. The 22 neighboring genes of one of those metabolites, kynurenine, showed significantly enriched binding sites for this transcription factor (p-value = 3.79 × 10^−09^, 73% of the 22 promoter sequences contained at least one occurrence of the motif; see Fig 5C and S7 Table).

### Integration of clinical phenotypes identifies active modules

**Fig 6:**
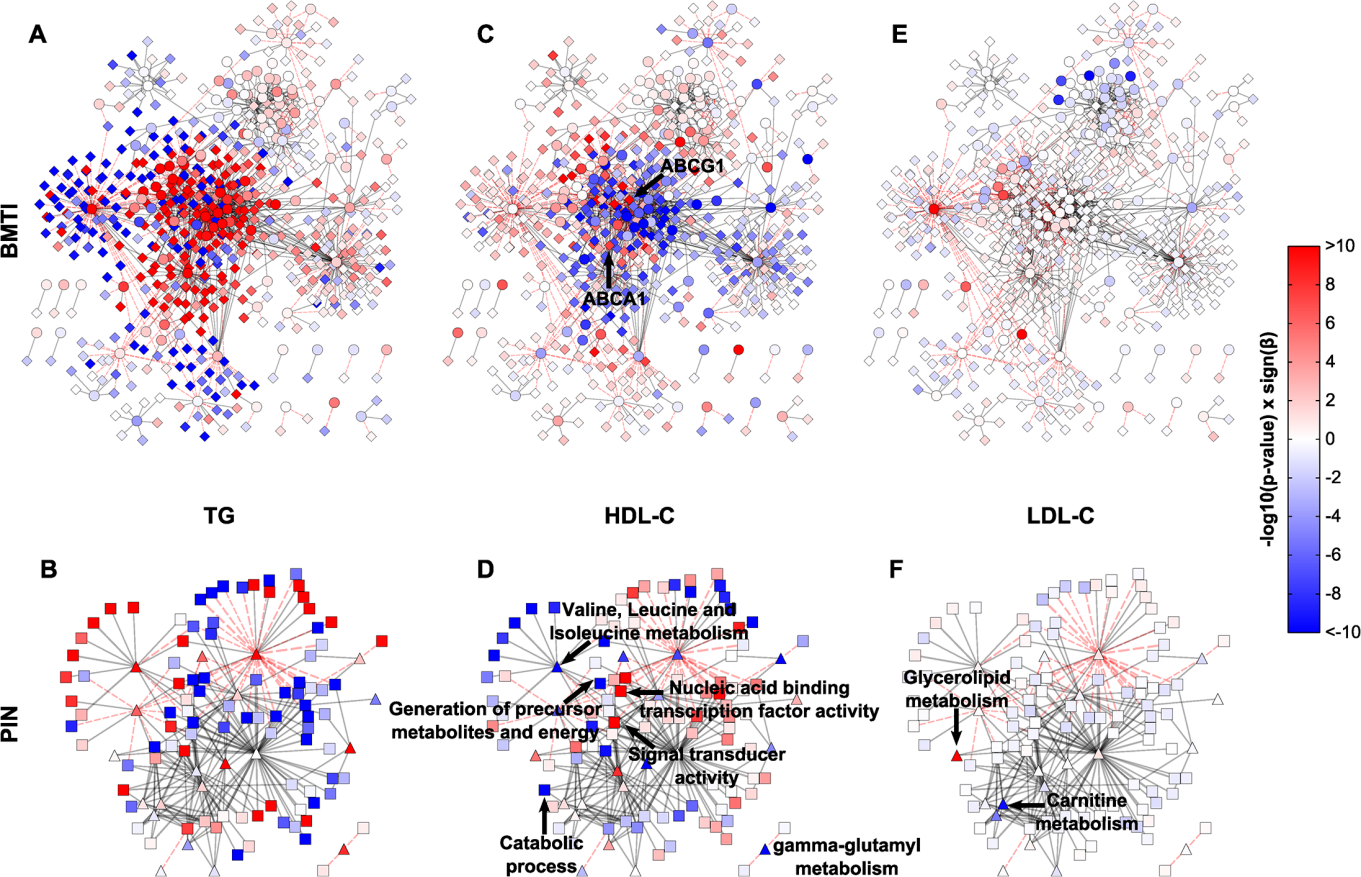
Intermediate clinical phenotype associations. Linear regression results of TG, HDL and LDL for each metabolite and transcript **(A, C, E)**, or pathway and GO term **(B, D, F)** were projected onto the respective networks. Blue colors indicate negative associations, while red colors represent positive associations to the respective phenotype. Color strength of the nodes encodes the-log10 p-value of the respective association. *β* denotes the regression coefficient and its sign represents the direction of the associations (positive or negative correlation).

As a final analysis step, we sought to use the BMTI and the PIN to infer novel insights into the molecular mechanisms and pathways underlying complex traits. To this end, we associated the nodes of both networks with intermediate clinical phenotypes (Fig 1E, Table 2). As already stated earlier, we chose the levels of HDL-C and LDL-C, as well as concentrations of blood triglycerides (TG), known risk factors for a variety of diseases. We performed multiple linear regression analyses with HDL-C, LDL-C and TG blood parameters as response variables and all 440 metabolites and 16,780 transcripts as explanatory variables. All models were corrected for sex and age. Statistical significance was defined by a Bonferroni adjusted p-value cutoff at 2.9 × 10^−6^ (*α* = 0.05). We then projected the −*log*10 transformed p-values from this regression as colors onto the corresponding nodes in the two networks. Similarly, the analysis was performed using aggZ-scores of pathways / GO terms as explanatory variables and mapped to the PIN (Fig 6 and S1 Dataset). Note that we presented similar approaches in the past for metabolomics-only networks [24,74].

**Table 2:** Characteristics of the KORA F4 study population.

Variable	
N (Male/Female)	712 (354/358)
	**Mean (sd)**
Age (years)	68.82 (4.31)
BMI (kg/m^2^)	28.87 (4.56)
HDL cholesterol (mg/dl)	55.80 (13.95)
LDL cholesterol (mg/dl)	140.60 (35.97)
Triacylglycerides (mg/dl)	132.64 (75.70)

N, number of individuals; BMI, body mass index; HDL, high density lipoprotein; LDL, low density lipoprotein; sd, standard deviation.

In total, regression analyses yielded 233 (54 metabolites, 179 mRNAs), 28 (28 metabolites, 0 mRNAs) and 1,124 (49 metabolites, 1,075 mRNAs) statistically significant associations for HDL-C, LDL-C and TG, respectively. Of those associations, 64%, 28% and 25%, were contained in the BMTI, respectively (see S9 Table for a complete list of associations). We only observed few LDL-C metabolite associations, which can be mainly summarized in the “Glycerolipid metabolism” and “Carnitine metabolism”, while none were observable for the transcripts (Fig 6E and 6F, S9 Table). We will therefore focus on network associations for HDL-C and TG in the following.

Examination of the networks for HDL-C and TG revealed localized regions of similar associations, which reflect potentially co-regulated modules (Fig 6A and 6C). Interestingly, when compared to each other, there appeared to be an antagonistic pattern of associations for HDL-C and TG, which is in accordance with an overall negative correlation of the two traits (*ρ* = −0.53). This opposing pattern also holds for the categorical networks (Fig 6A–6D and S10 Table). To confirm this observation statistically, we utilized an approach to compare the different networks suggested by Floegel *et al*. [74]. Basically, we calculated the Spearman correlation of the association measures between the different clinical traits. This resulted in a strong negative correlation between the BMTI-HDL-C and the BMTI-TG network (*ρ* = −0.84) which was even more pronounced between the PIN-HDL-C and PIN-TG networks (*ρ* = −0.94, S9 and S10 Figs). A similar pattern of opposing associations between HDL-C and TG phenotypic traits was already described in previous studies, which suggested a pleiotropic, heritable relation between the two lipid and lipoprotein measures, potentially regulated by a common, complementary mechanism [13,75].

In the following, we will discuss exemplary pathway mechanisms identified in the phenotype networks. ABCG1 and ABCA1, known constituents of the reverse cholesterol transport necessary for the proper formation of plasma HDL-C [55], were positively correlated with HDL-C (p-value = 4.37 × 10^−12^ and p-value = 2.92 × 10^−8^, respectively). At the pathway level, processes like “generation of precursor metabolites and energy” or “catabolic process” are negatively associated with HDL-C, while “nucleic acid binding transcription factor activity” and “signal transducer activity” are positively associated (Fig 6D). An inverse pattern can be seen for TG, where positive associations predominate and processes like “generation of precursor metabolites and energy” or “catabolic process” are strongly positively associated (Fig 6A and 6B).

Given the known association between HDL/TGs and branched-chain amino acids [57,76], we investigated the phenotypic networks to further examine this metabolic class. First, we examined the edge between isoleucine and ABCG1 within the BMTI-HDL-C network. As already mentioned, ABCG1 was strongly positively associated to HDL-C levels, while we found that isoleucine was significantly negatively associated to the concentration of HDL-C (*β* = −4.30, p-value = 5.80 × 10^−19^). Moreover, gamma-glutamyl variants of BCAAs belonging to “gamma-glutamyl metabolism” (*β* = −4.84, p-value = 3.15 × 10^−14^) and “Valine, leucine and isoleucine metabolism” (*β* = −4.66, p-value = 9.17 × 10^−11^) displayed profound negative associations to HDL-C (Fig 6D and S10 Table), further validating a connection between HDL-C and BCAA metabolism. For triglycerides, we observed an inverse relationship with BCAAs and BCAA-related pathways (Fig 6B, S9 and S10 Tables).

## Discussion

We constructed a global network model across two levels of biological information by integrating cross-sectional omics data from a large-scale population cohort. The dataset was based on circulating metabolites from plasma and transcriptional variation derived from whole blood. This analysis exploited the naturally-occurring variation caused by genetic variation, environmental and behavioral influences from a natural population over multiple layers of organization. Such an approach was recently referred to as ‘systems genetics’, enabling the systematic exploration of information flow between the different biological scales [32].

As mentioned in the introduction, blood is a heterogeneous tissue containing a series of distinct cell-types. In this study, we utilized whole blood transcriptomics data from unsorted cells, leading to a complex mixture of transcriptional signals in the transcriptome dataset [36]. Similarly, the levels of circulating metabolites are strongly influenced by metabolically active organs [31], but also by metabolites from blood cells and those taken up from the environment. The comparison to known cell-type specific markers further suggested that a substantial amount of the signals are derived from specific blood cells. However, the analysis also showed that the majority of the BMTI contained transcripts are not assigned to any cell-type. Thus, we assume that the metabolite-mRNA associations captured in the BMTI mainly reflect cell-type unspecific processes involved in the fundamental maintenance of cellular function, besides some processes specifically related to immune functions.

Independent replication of the BMTI edges was investigated using data from the DILGOM study. Out of 211 possible associations, we were able to replicate 29% at a nominal significance and 18% after multiple testing correction (FDR<0.05). This relatively low number of replicated associations might have various reasons. For example, 1) The DILGOM study used an NMR-based metabolomics platform in contrast to the mass spectrometry-based methodology used in KORA. 2) The smaller sample size of the DILGOM study might limit the power to detect existing associations between metabolites and transcripts. 3) Differences in laboratory procedures and protocols or the population structure can affect replication across cohorts. Future high-powered studies with more similar measurement platforms can further address the stability of metabolite-transcript correlations across studies.

A comprehensive analysis of the strongest associations between transcripts and metabolites clearly revealed biologically reasonable relationships, such as signaling and transport mechanisms. Many identified associations, e.g. between cortisol and *DDIT4* or between *SLC25A20* and multiple long chain fatty acids, were in consent with known signaling or metabolic pathways. Others support and extend results from previous studies. As one example, nearly all transcripts included in the lipid-leukocyte (LL) module identified by Inouye *et al* [29] were among the top scoring association pairs. For instance, we were able to confirm associations between *CP3A*, *FCER1A*, *GATA2*, *HDC*, *MS4A2*, and *SLC45A3*, core genes of the LL module, and leucine, isoleucine, and several lipids (see S1 Table). In addition, we found associations which, to the best of our knowledge, have not been described before. These include associations between 1-monoolein and *GATA2*, a key regulator of hematopoiesis, or *SLC45A3*, a known diagnostic marker for prostate cancer [77]. The identified associations extend the current knowledge about the connection between system-wide metabolism and immunity-related pathways.

Causal inference of the metabolite-mRNA associations using Mendelian randomization yielded no statistically significant results. There are various possible reasons for this negative outcome. First, there might be no causal effect in either direction between the investigated transcripts and metabolites. Besides that, the lack of significant findings could also be caused by the limitations of Mendelian randomization. For instance, MR is known to require large numbers of samples to detect true causal relationships, and the power in our study (n = 712) might have been too low [58]. We therefore decided to leave a more detailed discussion and analysis of causal effects to future, high-powered studies.

Comparison of the blood metabolome-transcriptome interface with the most recent human genome-scale metabolic reconstruction [33] allowed to assess whether transcript-metabolite correlations also recapitulate known biochemical reactions at a systematic level. We were able to show that strong associations between enzymes (represented by their respective transcripts) and metabolites are significantly accumulated at shorter pathway distances (Fig 3B and 3C), which is consistent with previous studies [60,78,79]. Further functional characterization identified transport, energy, lipid and amino acid subsystems to be predominately present at short pathway distances (Fig 3D and S5 Fig). This observation may reflect metabolic proximity through the uptake of metabolic nutrients by metabolically active blood cells. For instance, in our analysis we found signatures for all three major sources for energy production: lipids, proteins (in terms of amino acids) and carbohydrates indicating an active use of fuel molecules for energy generation by the blood cells.

Our model-based analysis has several limitations. Obviously, any such analysis is heavily dependent on the quality of the underlying metabolic reconstructions, which are still far from being complete [80]. This incompleteness, together with a prevalent inconsistent nomenclature of metabolites allowed us to map only 121 out of 254 measured metabolites onto the metabolic network model. Another limitation is the incomplete coverage of the metabolome, which is owed to the capabilities of currently available technologies. In this study we used measurements of 440 metabolites, which corresponds to just ~10% of the estimated human serum metabolome [81]. Nevertheless, we believe that despite incomplete pathway mappings, our observations further indicate that combined metabolomics and transcriptomics data from human blood reflect reaction signatures of system-wide biological processes.

To further functionally characterize the blood metabolome-transcriptome interface at a global level, we developed a network approach based on functional annotations. To this end, we aggregated z-score transformed measurements of metabolites and transcripts into their corresponding metabolic pathways and gene ontology categories, respectively. This approach allowed us to calculate correlation values between different functional categories, rather than between single metabolites and transcripts only. From these associations, we generated a pathway interaction network (PIN) of associated metabolic pathways and Gene Ontology terms, substantially reducing the complexity of the original network and thus facilitating functional interpretations. Detailed inspection of the PIN revealed that correlating nodes resembled not only signatures of well-known biological processes, like the carnitine shuttle, but also suggested novel interactions such as a crosstalk between monoacylglycerols and immune system processes. Taken together, the pathway interaction network enabled us to verify and elevate observations from the single reaction level (see model-based analysis) onto a pathway level. Moreover, we are now able to explore associations between biological processes/pathways across different biological scales including those that are not necessarily covered by metabolism, such as signaling or transcriptional processes.

Given the high interconnectivity of the BMTI and the PIN, we asked whether these associations contain information about regulatory interactions across the different metabolite classes and pathways. Enrichment analysis of transcription factor binding sites in the promoter regions of the genes contained in our network identified regulatory signatures for transcripts associated to the same metabolite, which are additionally largely shared between metabolites belonging to different metabolic pathways (Fig 5, S7 and S8 Figs). There is a series of possible explanations for this observation. On the one hand, our findings could indicate that single metabolites/transcripts are fulfilling multiple roles, thus sharing several biochemical pathways. On the other hand, it might reflect regulatory interactions operationally linking different metabolic pathways. In depth investigation of 12 transcription factors included in the BMTI additionally revealed two “triad” network motifs between transcription factors *BCL6* and *NR4A2*, their target genes and the metabolites methionine and kynurenine, respectively. Remarkably, in a study conducted on mice fed a methionine and choline deficient diet, a significant reduction in the expression of *BCL6* was observed [82]. It is widely known that metabolites can act as intermediates in cellular signaling, thereby also regulating gene expression, and together with our findings we suggest that characteristics of metabolic regulation are captured in the BMTI. However, from a correlation network, the detection of an association between a metabolite and a transcript does not necessarily imply a regulatory relationship nor can a conclusion be drawn about the directionality of the relationship. Yet, a combined analysis might offer the opportunity to identify novel molecular mechanisms behind cellular regulation that need to be validated further by experimental evidence.

Besides transcriptional regulation mediated by TFs, a substantial fraction of transcripts are expected to be regulated by epigenetic processes [83]. Comparing 1,350 reported methylation-metabolite associations from a recent epigenome-wide association study [31] with our results surprisingly revealed only a single overlapping hit: X-03094 and the MAN2A2 transcript correlated in our study and also displayed a comparable methylation-metabolite association in the EWAS study. This sparse overlap could be explained by a phenomenon termed “phenotypic buffering” [32], where effects in one organizational layer (e.g. epigenetics) are not detectable anymore on the next layer (e.g. transcriptomics). A detailed explanation of this observation is beyond the scope of the present paper and needs further investigation.

Further following the scheme of a systems genetics approach, we integrated the two identified networks with intermediate clinical trait data. To this end, we investigated the relationships between changing levels of HDL-C, LDL-C and TG and all measured metabolites and transcripts, metabolic pathways and GO terms (Fig 6). A similar study already described an association between a gene-module derived from whole blood transcriptomics data and circulating lipid parameters including apolipoprotein B (APOB), HDL-C and triglycerides (TG) from a Finnish population cohort [29]. Our systematic analysis identified a large number of metabolites, transcripts, metabolic pathways, and functional GO categories that are all associated with the levels of circulating lipids. These findings further strengthen the assumption of a close link between system-wide metabolism, reflected by circulating metabolites and clinical lipid markers, and intracellular gene regulatory processes of blood cells. In addition, an opposite pattern between HDL-C and TG associations (Fig 6A–6F) was observed from the phenotype networks which supports a previously suggested antagonistic regulation of both clinical traits [75,84]. However, the precise molecular mechanism behind this regulation is not entirely known, and our results might provide a basis for future studies to gain novel insights into the regulatory mechanisms of intermediate physiological phenotypes.

Combining results from all analysis steps allows for novel hypothesis generation. For example, for the well-known interactions between HDL-C, TG and BCAAs [57,76], we discovered a potential regulatory pattern on different biological scales. In our first analysis step, we identified a strong negative association between the branched-chain amino acid isoleucine and ABCG1, a major constituent of lipid homeostasis and cholesterol metabolism [55,85]. Second, at a more global level, the phenotype networks revealed an inverse association between HDL-C and TG, and also between HDL-C, TG and BCAAs (BCAAs are positively associated to TG, negatively to HDL-C, see S9 Table). Third, in the TFBS enrichment analysis we were able to identify a clear regulatory signature of SREBPs in the vicinity of BCAAs, which are known to regulate cholesterol metabolism, indicating a potential coregulation between BCAAs and cholesterol metabolism at the transcriptional level. Interestingly, a combined study using cultured hepatocytes in a branched-chain amino acid-rich medium and obese mice showed that BCAAs directly induce the expression of *SREBP1C* which leads to hypertriglyceridemia, further supporting the suggested regulatory cross-link between HDL-C, TG and BCAAs [76]. This link is of particular interest since all three molecular traits have been associated to various diseases such as coronary artery disease, obesity and diabetes type II [86–88] and our observations might contribute to further decipher their underlying mechanisms.

In summary, our study highlights the potential of a systems genetics approach for understanding interactions across multiple biological scales – in this case circulating metabolites and blood cellular gene expression—and how those insights can be used to generate novel hypothesis on mechanisms underlying common diseases.

## Materials and Methods

### Population cohort and data acquisition

The Cooperative Health Research in the Region of Augsburg (KORA) study is a series of independent population-based epidemiological surveys and follow-up studies of participants living in the region of Augsburg, southern Germany [89,90]. In this paper, cross-sectional data from 712 participants of the KORA F4 population cohort was used for whom metabolite concentration, gene expression data and genotyping information were available. This subpopulation contains combined fasting serum metabolomics and whole blood transcriptomics measurements of 354 males and 358 females aged 62–77 years (mean 68.82 ± 4.31). All participants are residents of German nationality identified through the registration office and written informed consent was obtained from each participant. The study was approved by the local ethics committee (Bayerische Landesärztekammer). Detailed descriptions of blood sample acquisition and experimental procedures for the metabolomics and transcriptomics data, and clinical trait measurements can be found in [59,91–93]. Briefly, metabolic profiling was performed by Metabolon, Inc. using ultrahigh-performance liquid-phase chromatography and gas-chromatography separation, coupled with tandem mass spectrometry. In total, 517 serum metabolites were measured, thereof 293 with known chemical identity and 224 unidentified metabolites (“unknowns”). All identified metabolites were assigned to one out of eight *superpathways* and one out of 61 *subpathways* by Metabolon, Inc., representing two different levels in the metabolic pathway classification hierarchy (see S5 Table for a full list of annotations). Gene expression profiling was performed using total RNA extracts from whole blood samples on Illumina Human HT-12 v3 Expression BeadChips. Genotyping was carried out using the Affymetrix GeneChip array 6.0. A detailed description of the experimental procedures and preprocessing of the genetic data can be found in [92].

Replication of the significant metabolite- mRNA associations identified in the KORA dataset was carried out with the Finish DILGOM cohort dataset which included whole blood NMR metabolomics data as well as transcriptomics data for 518 individuals. A detailed description of the sample acquisition as well as data preparation can be found in [13,29].

### Data preprocessing and quality control

To ensure data quality, metabolites with more than 50% missing values were excluded, leaving 440 metabolites (254 knowns and 186 unknowns) for further analysis. The remaining metabolite concentrations were log-transformed, since testing for normality indicated that for most cases the log-transformed concentrations were closer to a normal distribution than the untransformed values [23]. For gene expression arrays, quality control and imputation of missing values of the raw intensities was performed as described in [94]. Briefly, the initial preprocessing of the raw intensity data was done with GenomeStudio V2010.1. Raw probe level data was then imported to R and further preprocessed by log transformation and quantile normalization using the ‘lumi’ package [95] from the Bioconductor open source software (http://www.bioconductor.org). To account for technical variation, gene expression intensities data were adjusted for RNA amplification batch, RNA integrity number and sample storage time. Only probes with the annotation flag QC_COMMENT “good” as provided in the updated Illumina Human HT-12 v3 BeadChip annotation file were considered for analysis [94]. In addition, probes mapping to gonosomal chromosomes were removed. Out of 48,803 probes on the Illumina Human HT-12 v3 array, 24,818 passed these filtering criteria.

### Correlation network generation

The metabolite-transcript interface was constructed based on Spearman’s correlation coefficients between the concentrations of all possible metabolite-transcript pairs (24,818x440) across the individuals of the study cohort. Correlation calculation was performed separately for each variable pair, only considering samples without missing values for the metabolites. Statistical significance of correlations was determined at an FDR of 0.01 [96], corresponding to an absolute correlation value of 0.1816 and an adjusted significance level of 1.07 × 10^−06^. To get a unique network node per gene, redundant probes matching the same gene were removed. One representative probe per gene was chosen based on the maximum correlation strength to any metabolite, leaving 16,781 unique probes for subsequent analysis. It has to be noted that the applied significance level was still calculated on the whole dataset (including multiple matching probes per gene) to properly account for multiple testing. Network density was calculated as described in [35]. More precisely, for a stepwise increasing correlation threshold, the ratio between the total number of observed edges and all possible edges was calculated. Significant correlations between metabolites and transcripts were visualized as a bipartite graph using yEd graph editor (yWorks GmbH, Tuebingen; http://www.yworks.com).

### Tissue/cell-type specificity

BMTI genes were mapped to three published lists of tissue- and cell-specificity based on gene expression microarrays from purified cells or tissues. The first two marker gene lists were taken from Palmer et al. [36], who defined markers for B-cells, CD4+ T-cells and CD8+ T-cells, lymphocytes and granulocytes, and from the HaemAtlas as generated by Watkins et al. [37], who reported markers for CD19+ B-cells, CD4+ T-cells and CD8+ T-cells, CD14+ monocytes, CD56+ NK cells, CD66b+ granulocytes, erythroblasts and megakaryocytes. The third marker list was downloaded from the CTen website: http://www.influenza-x.org/~jshoemaker/cten/db_info.php and comprised markers for 84 different human tissues/cell types [38]. The three lists together with the analysis results are provided in S3 Table.

### Mendelian randomization

Estimation of causal effects within the BMTI was performed using a Mendelian randomization (MR) approach [58]. A total of 224 candidate SNPs reported as lead association signals at genome-wide significance in two recent GWAS studies for 16 metabolites and 186 mRNAs (BMTI contained) were preselected as instrumental variables[23,59]. To ensure the validity of the instrumental variables, only candidate SNPs that showed a significant association with a trait (metabolite or gene expression level) at an FDR of 0.05 in our data were considered for further analysis (32 SNPs were removed). Associations between SNPs and traits were assessed using linear regressions with age and sex as covariates. To further avoid potential confounding, all candidate SNPs were checked for pairwise linkage disequilibrium using the SNiPA tool [97]. None of the remaining 192 SNPs were in LD. Based on the metabolite-mRNA edges in the BMTI, 550 SNP-metabolite(Met)-mRNA and SNP-mRNA-Met sets were defined, covering 44% of all edges contained in the BMTI. Causal relationships SNP→Met→mRNA and SNP→mRNA→Met were estimated, i.e. whether changes in the metabolite level cause changes in the transcript level and vice versa. Causal effects of both models were calculated using the Wald ratio method [98]:

$${\hat{\beta }}_{Met\to mRNA}=\frac{{\hat{\beta }}_{SNP\to mRNA}}{{\hat{\beta }}_{SNP\to Met}}\;\text{and}\;{\hat{\beta }}_{mRNA\to Met}=\frac{{\hat{\beta }}_{SNP\to Met}}{{\hat{\beta }}_{SNP\to mRNA}},$$

where ${\hat{\beta }}_{Met\to mRNA}$ and ${\hat{\beta }}_{mRNA\to Met}$ are the causal effects, and ${\hat{\beta }}_{SNP\to mRNA}$ and ${\hat{\beta }}_{SNP\to Met}$ are regression coefficients of the respective mRNA or metabolite levels on SNPs, under a simple linear model with age and sex as adjustment variables. 95% Confidence intervals and p-values of the causal effects were calculated by sample bootstrapping with 10,000 repetitions. Q-values were calculated to control the false discovery rate (FDR). Summary information for the utilized SNPs together with detailed results of the MR approach can be found in S4 Table.

### Metabolic pathway model and distance calculation

Metabolic reactions were extracted from the consensus metabolic reconstruction “Recon 2”, v. 02 available at http://humanmetabolism.org as of October 2013 [33]. Compartmental information was removed by merging shared nodes and reactions between different compartments. To avoid potential biologically meaningless shortcuts between network nodes, co-factors and currency metabolites were excluded from the metabolic network prior to the distance calculation (see S5 Table for a full list of removed metabolites). Measured metabolites and transcripts were mapped onto the corresponding network nodes based on KEGG IDs or HMDB identifier for metabolites, and Entrez Gene IDs for transcripts. Distances between all mapped pairs of metabolites and transcripts were defined as the shortest path in the network, i.e. the minimal number of reaction steps between them. For instance, a distance of zero between a transcript and metabolite indicates that the metabolite is a direct reactant of the reaction catalyzed by the particular enzyme encoded by the transcript. A distance of one indicates that the enzyme-encoding transcript catalyzes a directly connected reaction, which takes a product of the particular metabolite as input, and so on. A distance of infinity (Inf) was assigned if the respective metabolite and transcript were disconnected in the pathway network. Moreover, a “not mapped” (NM) distance was assigned if either the metabolite or the transcript could not be mapped to Recon 2. Note that the network was treated as undirected, i.e. all reaction directions were ignored.

### Annotations, aggregated z-scores and construction of pathway interaction network

Functional annotations were retrieved from two different sources. For transcripts, the generic GO Slim catalogue was downloaded from Gene Ontology (GO, http://www.geneontology.org/GO.slims.shtml). Generic GO Slim is a broad and non-redundant subset of all Gene Ontology terms consisting of 148 unique terms covering all three GO domains (cellular component, molecular function and biological process; [99]). The three root terms *cellular component*, *molecular function* and *biological process* and terms with no annotations for any of the 16,781 transcripts were removed, resulting in 140 terms for further analysis. For metabolites, the *subpathway* annotations were used (see above). Metabolic pathways (MP) with less than two metabolites were excluded from the analysis, leaving 48 metabolic pathways.

To aggregate the components belonging to a specific annotation term and to derive a score for each of these functional categories, the average of the associated z-score normalized gene expression profiles or metabolite concentrations was calculated according to

$${\text{aggZC}}_{\text{j}}=\frac{1}{\left|C\right|}\sum_{i\in C}Z_{i,j}$$

where C corresponds to a metabolic pathway or GO term, *i* enumerates all members in this set, and *Z*_*i*,*j*_ is the z-score of the gene/metabolite with index *i* in sample *j*. Spearman’s rank cross-correlation between the aggZ-Scores of all possible GO-MP combinations was then calculated (note that Pearson correlation yielded similar results, see S11 Fig). Since it is known that many biological processes include distinct branches often fulfilling complementary tasks controlled by mutual regulation, a consideration of all pathway members simultaneously could obscure the calculation of the aggZ-Score. A similar problem might occur due to the generic property of the GO-terms or metabolite classes used here, often including functionally rather distinct molecules. To account for this, only those members of the two categories were considered for z-score calculations which share at least one mutual edge within the reconstructed network for the respective GO-MP combination (see S6 Fig for more details). Finally, significant associations between the functional annotation pairs were visualized as a bipartite pathway interaction network (PIN).

### Phenotype analysis

Linear regression analysis was performed with age and sex as covariates:

$$y=\beta_{0}+\beta_{1}*x+\beta_{2}*\text{age}+\beta_{3}*\text{sex}+\epsilon$$

where *y* is the concentration of HDL, LDL or TG over all individuals, *β*_0_ is the intercept, *β*_1–3_ are regression coefficients, *x* is a vector of expression/concentration values of a particular gene/metabolite and *ϵ* is a normally distributed error term. In the same way, the association of annotations (GO and MP) was tested with all three phenotypic traits using the aggZ-Score for the particular annotation of *x*. Note that for this analysis, aggZ-Scores were calculated only on those members of a particular annotation that are also contained in the BMTI. Each network node was then color-coded with the −log_10_(p-value) × sign(*β*_1_), where the p-value and *β*_1_ were derived from the linear regression with the respective metabolite, gene or annotation category. To assess statistical significance of the determined associations, a Bonferroni-corrected threshold of 0.05/(16,780 × 440) ≈ 2.9 × 10^−6^ was applied.

### Promoter analysis

To investigate regulatory signatures in the BMTI, an enrichment analysis of transcription factor binding sites was performed. Sets of input sequences were created from the neighbors of each metabolite with a degree ≥ 3 (at least 3 connected genes). Analogously, the pathway interaction network was used to construct sequence sets based on the neighborhood of a metabolic pathway node. For each set of input sequences, a separate search for overrepresented TFBS was performed with the sequences of all remaining genes as background model. Promoter regions (-2,000 bp to +200 bp relative to the TSS) and TSS positions of all genes were extracted from the UCSC database using the R package BSgenome.Hsapiens.UCSC.hg19 version 1.3.1. Position-specific weight matrices of the transcription factor binding motifs were taken from the vertebrate collection of the Jaspar database version 5.0 alpha [68]. Enrichment analysis was performed with the TFM-Explorer command line tool [100]. The p-value threshold to determine significance of the motifs in all input sets was set to 1.0 × 10^−7^ which lies in the recommended optimal range given the numbers of input sequences we used in this study (mean number of input sequences: 62) [101]. The authors showed that for a fixed false positive rate of 10%, the optimal p-value threshold was 1.0 × 10^−7^ for a dataset of 100 input sequences.

## Supporting Information

S1 Dataset The BMTI and PIN networks in two interactive formats. The .cys file can be viewed with http://www.cytoscape.org/; the .graphml file can be viewed with http://www.yworks.com/en/products/yfiles/yed/
(ZIP)

S1 Fig Scatterplot of Spearman and Pearson correlations for all metabolite-mRNA pairs in the dataset. Note that due to the high number of pairs (440x16780), only every 200^th^ correlation of the ordered list of correlation coefficients is plotted. We observe a high agreement between the two measures, with a correlation (of correlations) of r = 0.92.
(PDF)

S2 Fig Network density. The fraction of edges above cutoff against all possible edges, plotted as a function of the absolute correlation coefficient. Red dotted line represents the correlation cutoff used in this study (0.01 FDR).
(PDF)

S3 Fig Fractions of metabolite-transcript pairs with a given pathway distance. The first column (‘all finite’) contains mapped pairs with non-infinite distances. The second column (‘all mapped’) contains all pairs of metabolites and transcripts that could be mapped to the pathway network. The third column (‘all distances’) shows all pairs, indicating that the larger fraction of pairs cannot be mapped to the pathway network. The first row (‘significant’) only contains significantly correlating pairs of metabolites and transcripts, whereas the second row (‘all’) shows all pairs. The increase in fraction size from ‘all’ to ‘significant’ is assessed by a Fisher’s exact test and shown in Fig 3C in the main manuscript.
(PDF)

S4 Fig Scatter plots for an exemplary path of length 2. Note that this path corresponds to the one depicted in Fig 3A. The path describes the ACSL-catalyzed activation of stearate (18:0), followed by mitochondrial transport via the carnitine shuttle. The transport process is a two-step reaction including the attachment of carnitine by *Carnitine Palmitoyltransferase 1A* (*CPT1A*) at the outer mitochondrial membrane and subsequent internalization by carnitine-acylcarnitine translocase (*SLC25A20*). Upper triangle matrix indicates Spearman correlation coefficients. For direct substrate/product–enzyme pairs of this path we observe weak, insignificant associations ranging from *ρ* = 0.0016 to *ρ* = 0.13. In contrast, the strongest correlation was observed between stearate (18:0) and *SLC25A20* (*ρ* = 0.36, p-value = 2.02 × 10^−24^), which are two reaction steps apart.
(PDF)

S5 Fig Functional annotation of transcripts at a distance of 2. Similar to shorter distances, the strongest associations between metabolites and transcripts at a distance of two mainly resemble paths describing transport processes (28%) or those belonging to energy metabolism (oxidative phosphorylation 25%), amino acid metabolism (methionine and cysteine metabolism 11%, histidine metabolism 4%, glutamate metabolism 4%) and lipid metabolism (fatty acid oxidation 14%) besides others.
(PDF)

S6 Fig Pathway interaction analysis. The left-hand side of the flowchart shows an exemplary set of interactions between 4 transcripts and 4 metabolites, with annotations to two gene ontology terms and two metabolic pathways. For each GO-pathway pair we then determine the transcripts and metabolites which generate the connection between those two (middle panel). For example, GO1 and MP1 are connected through 3 transcripts and 2 metabolites, whereas GO1 and MP2 do not share any connection. Aggregated z-scores are then calculated for each pair based on the shared transcripts and metabolites to generate the pathway interaction network (left-hand side).
(PDF)

S7 Fig Matrix showing the overlap of enriched transcription-factor binding sites for all metabolites that are included in the BMTI and have at least one enriched site. On this fine grained level of single metabolites, several signatures of a shared regulation are observable, e.g. between amino acids and various other metabolites. Note that the color-scale is capped at 15, the maximum number of shared binding sites was 27 between histidine and X-03094.
(PDF)

S8 Fig Matrix showing the overlap of enriched transcription-factor binding sites for all super-pathways. On this coarse level, only few but highly significant shared regulatory signatures are observable. Note that the color-scale is capped at 15, the maximum number of shared binding sites was 4 between carbohydrate and peptide metabolism. The category “unassigned” includes all unknown metabolites.
(PDF)

S9 Fig Correlation of phenotype associations. Comparison of beta values from linear regression analysis. Each dot represents either a transcript or a metabolite in the BMTI. We observe a strong anti-correlation between HDL and TG associations, modest positive correlation between TG and LDL and a very weak anti-correlation for HDL and LDL.
(PDF)

S10 Fig Correlation of phenotype associations at pathway level. Comparison of beta values from linear regression analysis. Each dot represents either a GO term or a metabolic pathway in the PIN. Even more profound than for single transcripts and metabolites (S9 Fig), there is a strong anti-correlation between HDL and TG associations. The correlations for HDL and LDL as well as LDL and TG associations are comparable to the results from S9 Fig.
(PDF)

S11 Fig Comparison of Spearman and Pearson correlations of pathway scores. Similar to the results of transcripts and metabolites (S1 Fig), we observe high concordance of Spearman and Pearson correlation coefficients for the pathway scores (r = 0.99).
(PDF)

S1 Table All statistically significant associations between metabolites and transcripts. (XLSX)

S2 Table Replication using data from the DILGOM study. (XLSX)

S3 Table Cell type specific origins of BMTI transcripts. (XLSX)

S4 Table Mendelian randomization results. (XLSX)

S5 Table List of cofactors removed from Recon 2 and all measured metabolites together with available pathway annotations. (XLSX)

S6 Table All pairwise metabolic pathway – GO term associations. (XLSX)

S7 Table Enrichment of TFBS in promoter regions per single metabolite / metabolic pathway. (XLSX)

S8 Table Shared enriched TFBS between metabolites, subpathways and superpathways. (XLSX)

S9 Table Table containing the associations between HDL-C, LDL-C, TG and all metabolites/ transcripts. (XLSX)

S10 Table Associations between HDL-C, LDL-C, TG and all metabolic pathways/ GO terms. (XLSX)
