# Supplementary figures and images for: The Human Blood Metabolome-Transcriptome Interface
Source: PLoS Genet. 2015 Jun 18;11(6):e1005274. doi: 10.1371/journal.pgen.1005274 (PMC4473262; doi:10.1371/journal.pgen.1005274)

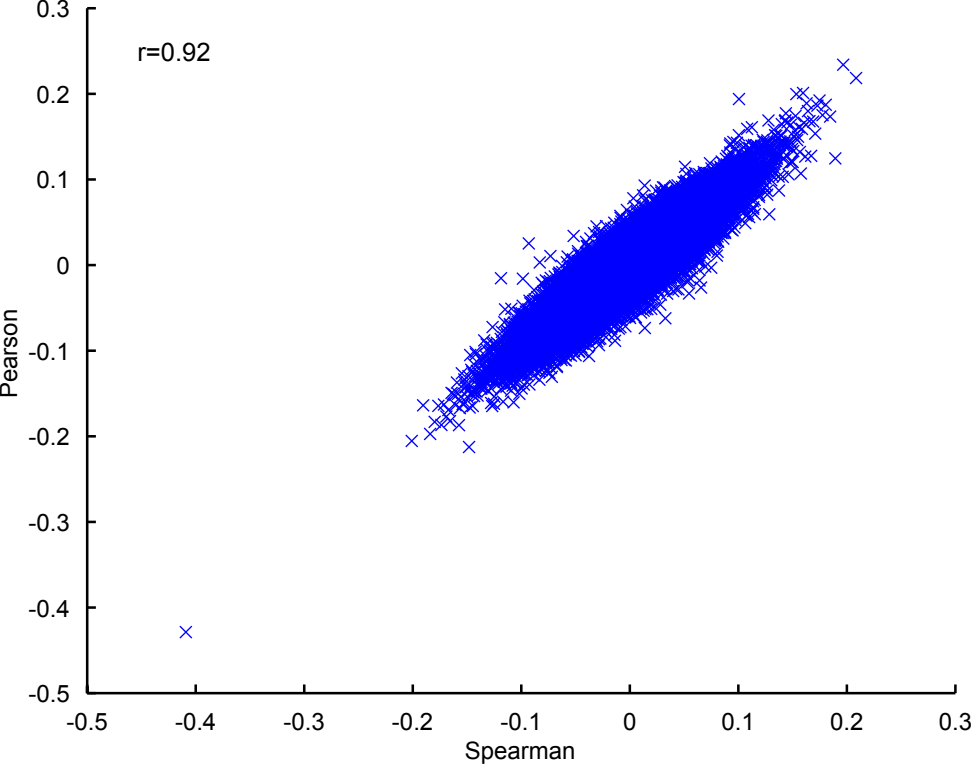

Supplement: S1 Fig — Note that due to the high number of pairs (440x16780), only every 200th correlation of the ordered list of correlation coefficients is plotted. We observe a high agreement between the two measures, with a correlation (of correlations) of r = 0.92. (PDF) [file pgen.1005274.s002.pdf]

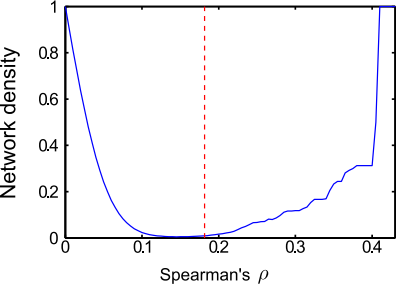

Supplement: S2 Fig — The fraction of edges above cutoff against all possible edges, plotted as a function of the absolute correlation coefficient. Red dotted line represents the correlation cutoff used in this study (0.01 FDR). (PDF) [file pgen.1005274.s003.pdf]

significant

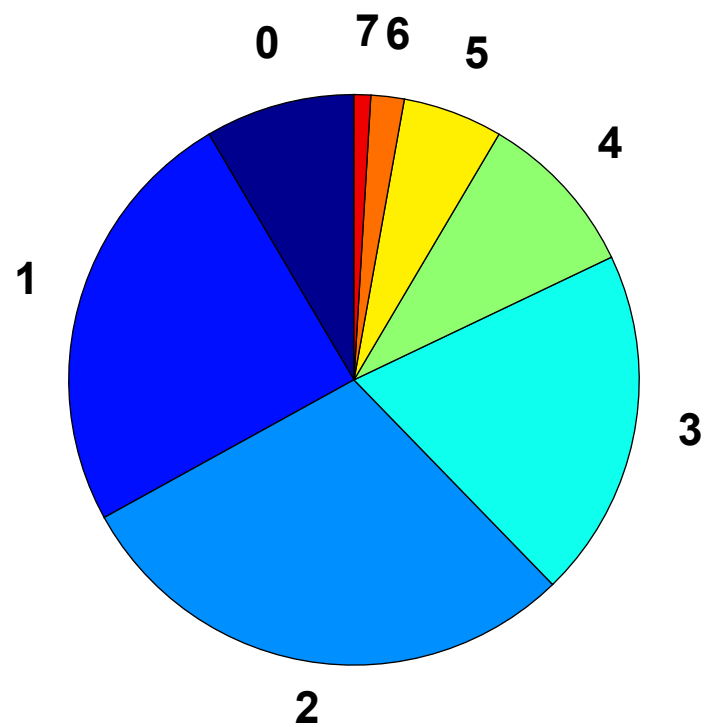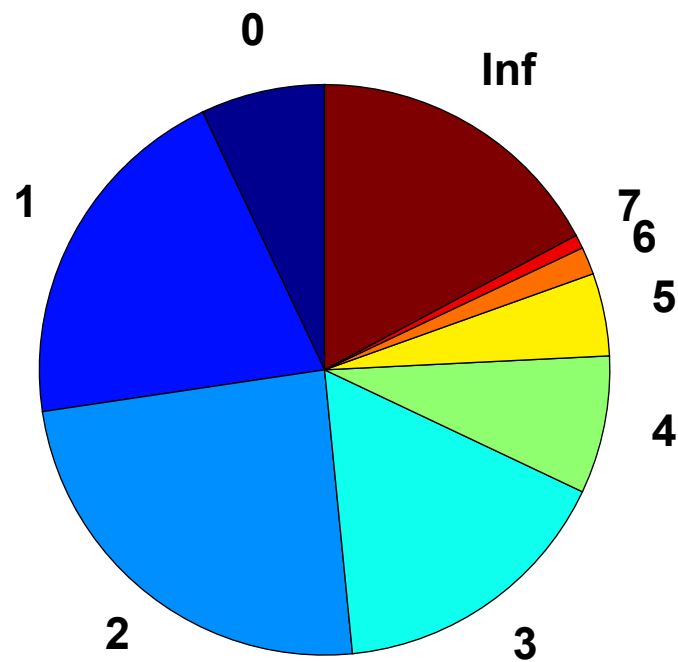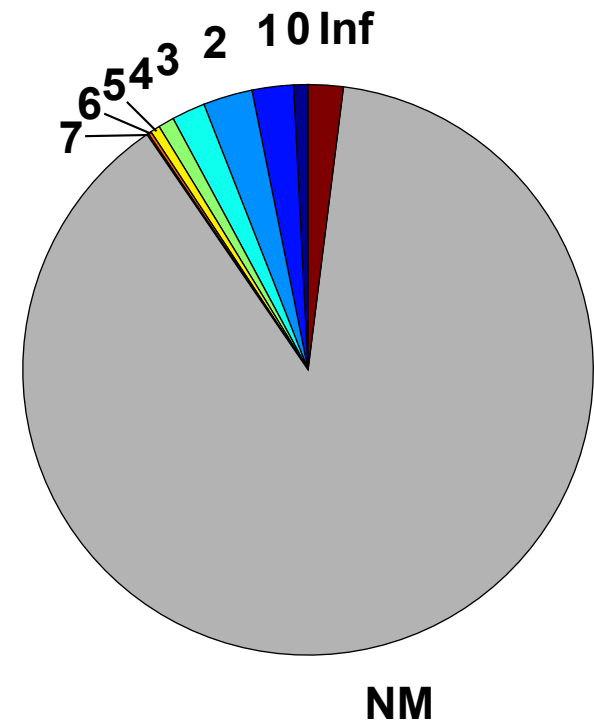

all

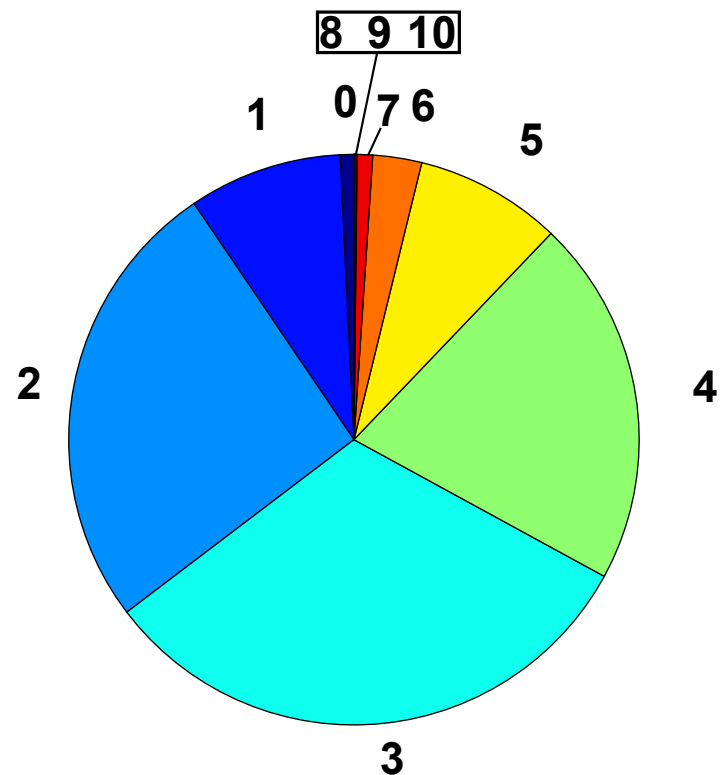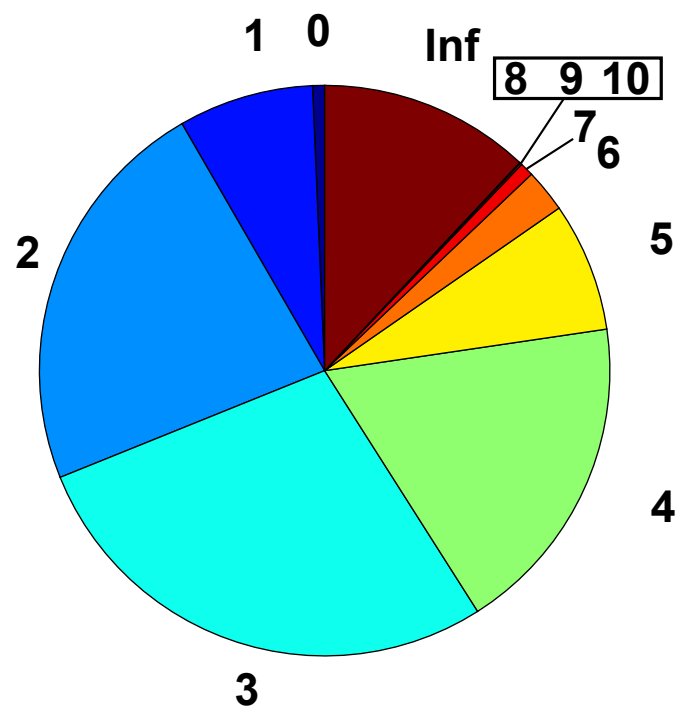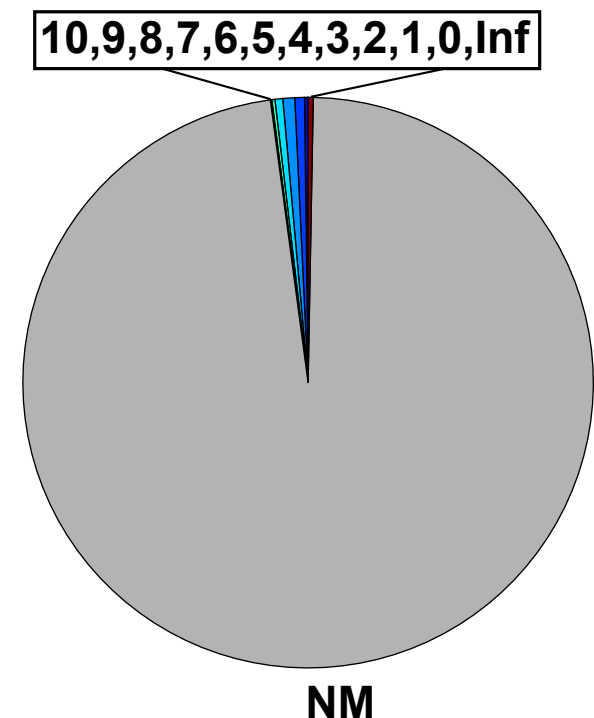

Supplement: S3 Fig — The first column (‘all finite’) contains mapped pairs with non-infinite distances. The second column (‘all mapped’) contains all pairs of metabolites and transcripts that could be mapped to the pathway network. The third column (‘all distances’) shows all pairs, indicating that the larger fraction of pairs cannot be mapped to the pathway network. The first row (‘significant’) only contains significantly correlating pairs of metabolites and transcripts, whereas the second row (‘all’) shows all pairs. The increase in fraction size from ‘all’ to ‘significant’ is assessed by a Fisher’s exact test and shown in Fig 3C in the main manuscript. (PDF) [file pgen.1005274.s004.pdf]

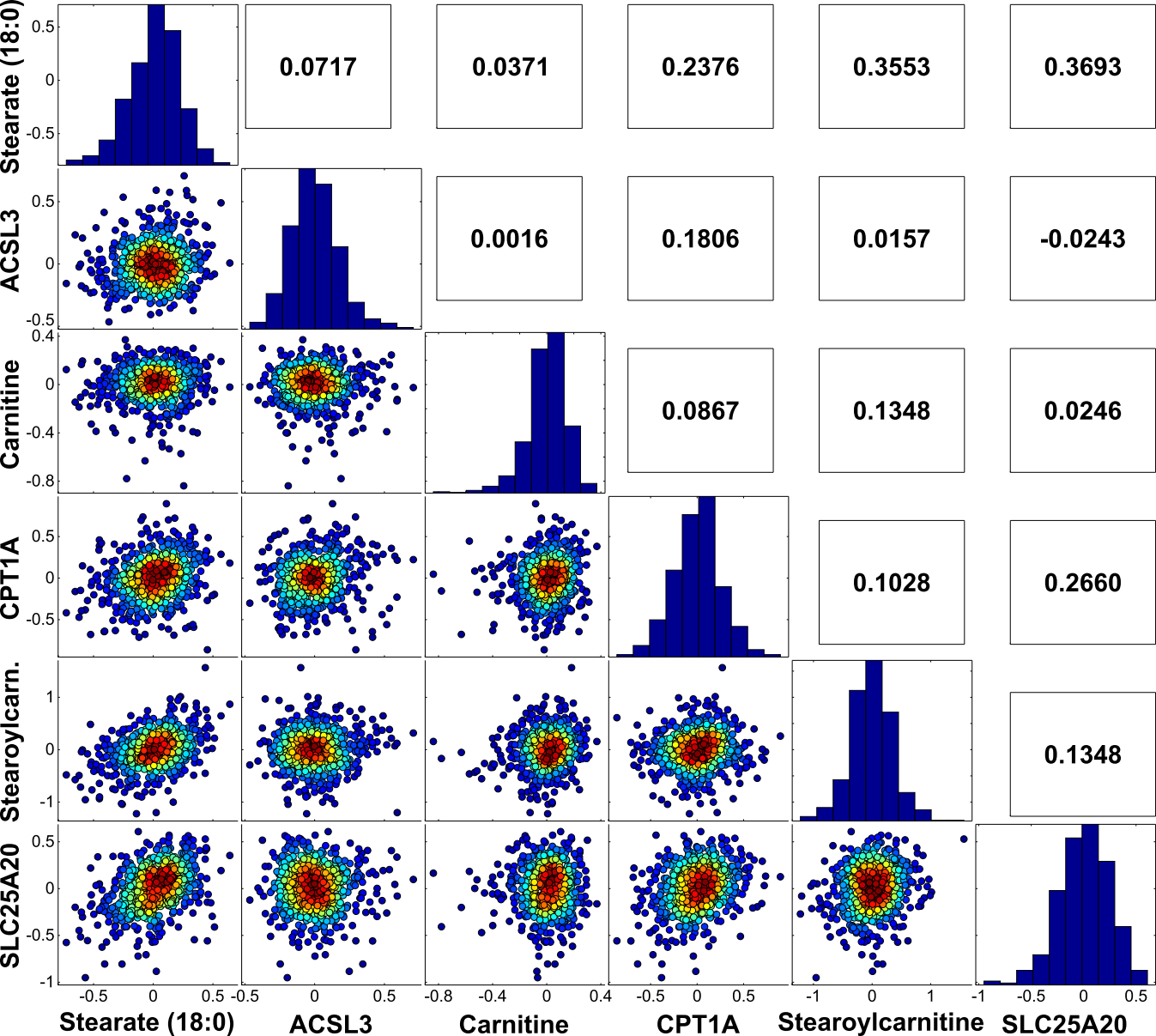

Supplement: S4 Fig — Note that this path corresponds to the one depicted in Fig 3A. The path describes the ACSL-catalyzed activation of stearate (18:0), followed by mitochondrial transport via the carnitine shuttle. The transport process is a two-step reaction including the attachment of carnitine by Carnitine Palmitoyltransferase 1A (CPT1A) at the outer mitochondrial membrane and subsequent internalization by carnitine-acylcarnitine translocase (SLC25A20). Upper triangle matrix indicates Spearman correlation coefficients. For direct substrate/product–enzyme pairs of this path we observe weak, insignificant associations ranging from ρ = 0.0016 to ρ = 0.13. In contrast, the strongest correlation was observed between stearate (18:0) and SLC25A20 (ρ = 0.36, p-value = 2.02 × 10^−24^), which are two reaction steps apart. (PDF) [file pgen.1005274.s005.pdf]

## distance 2

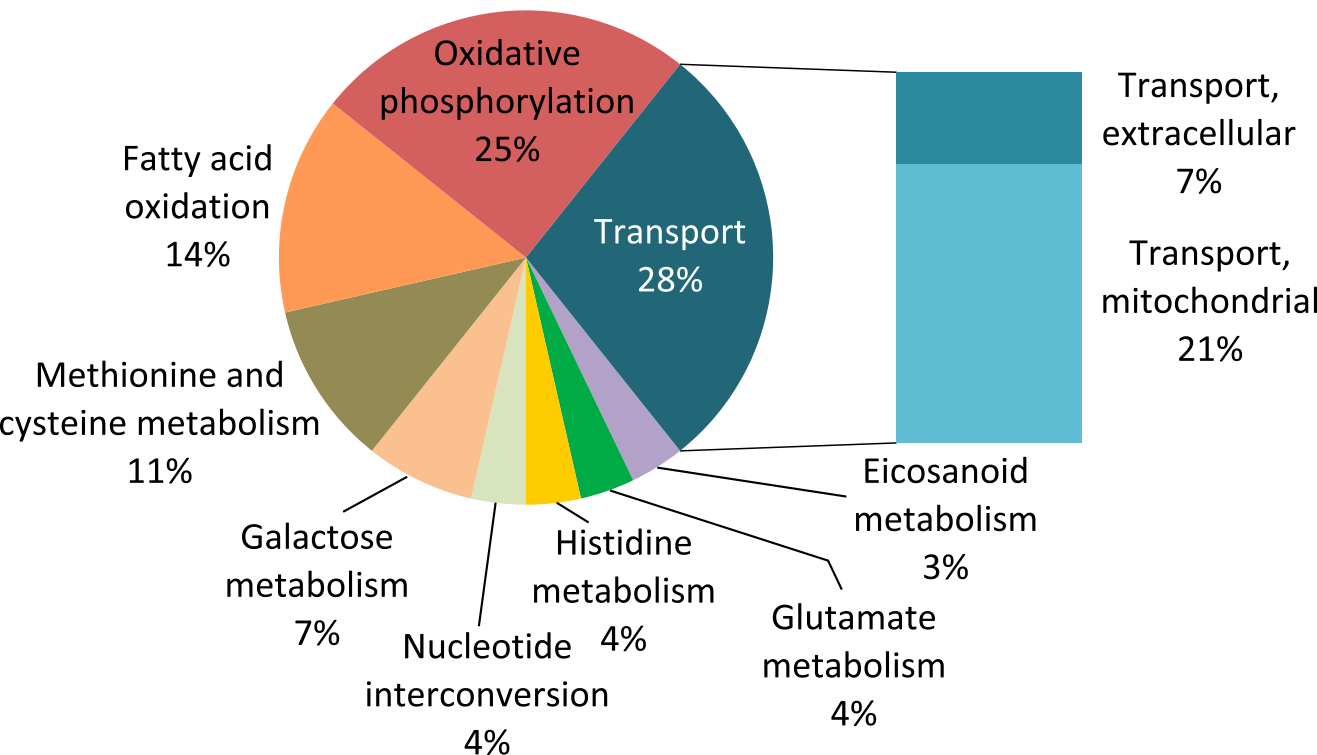

Supplement: S5 Fig — Similar to shorter distances, the strongest associations between metabolites and transcripts at a distance of two mainly resemble paths describing transport processes (28%) or those belonging to energy metabolism (oxidative phosphorylation 25%), amino acid metabolism (methionine and cysteine metabolism 11%, histidine metabolism 4%, glutamate metabolism 4%) and lipid metabolism (fatty acid oxidation 14%) besides others. (PDF) [file pgen.1005274.s006.pdf]

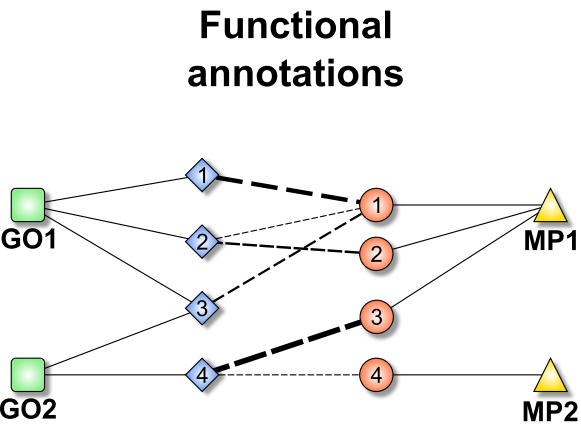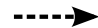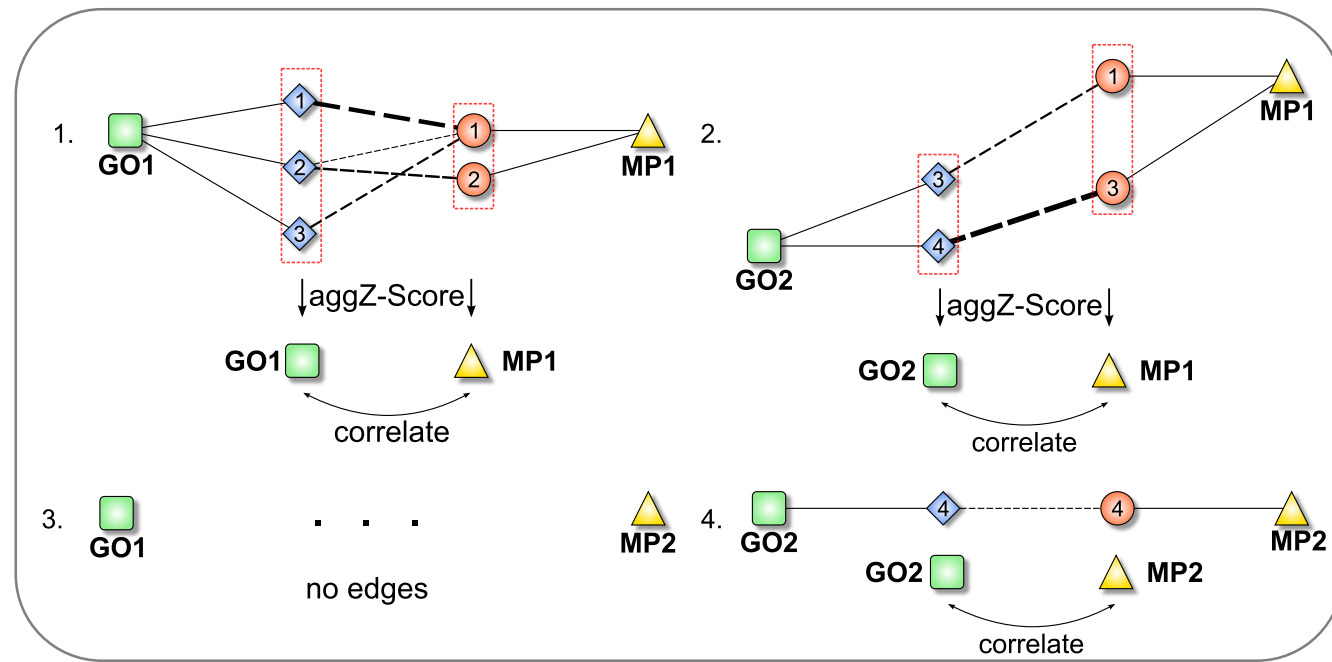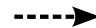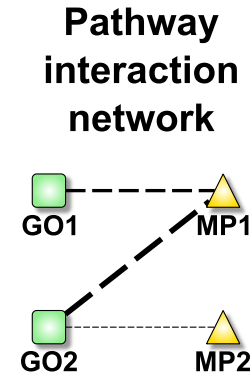

Supplement: S6 Fig — The left-hand side of the flowchart shows an exemplary set of interactions between 4 transcripts and 4 metabolites, with annotations to two gene ontology terms and two metabolic pathways. For each GO-pathway pair we then determine the transcripts and metabolites which generate the connection between those two (middle panel). For example, GO1 and MP1 are connected through 3 transcripts and 2 metabolites, whereas GO1 and MP2 do not share any connection. Aggregated z-scores are then calculated for each pair based on the shared transcripts and metabolites to generate the pathway interaction network (left-hand side). (PDF) [file pgen.1005274.s007.pdf]

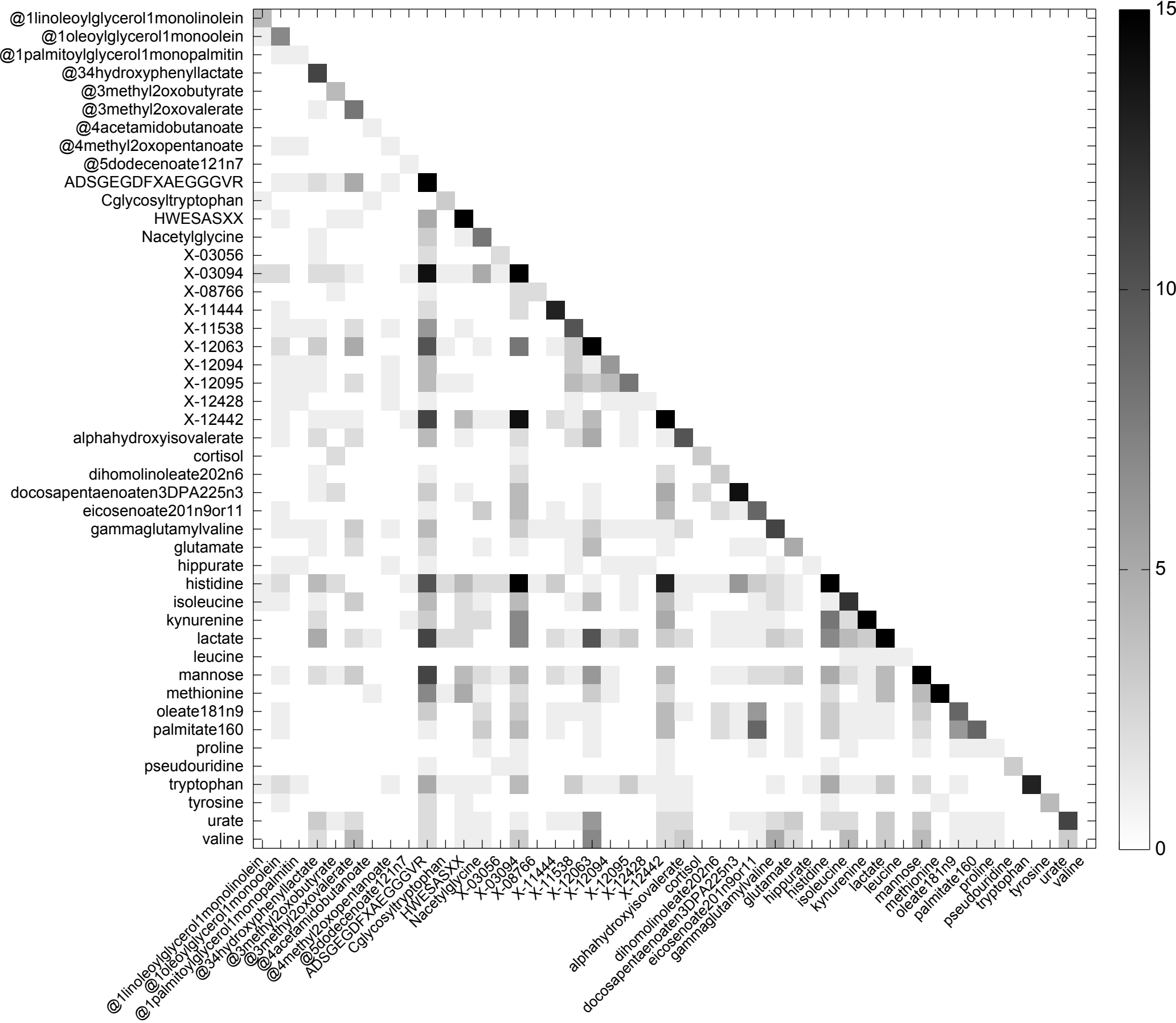

Supplement: S7 Fig — On this fine grained level of single metabolites, several signatures of a shared regulation are observable, e.g. between amino acids and various other metabolites. Note that the color-scale is capped at 15, the maximum number of shared binding sites was 27 between histidine and X-03094. (PDF) [file pgen.1005274.s008.pdf]

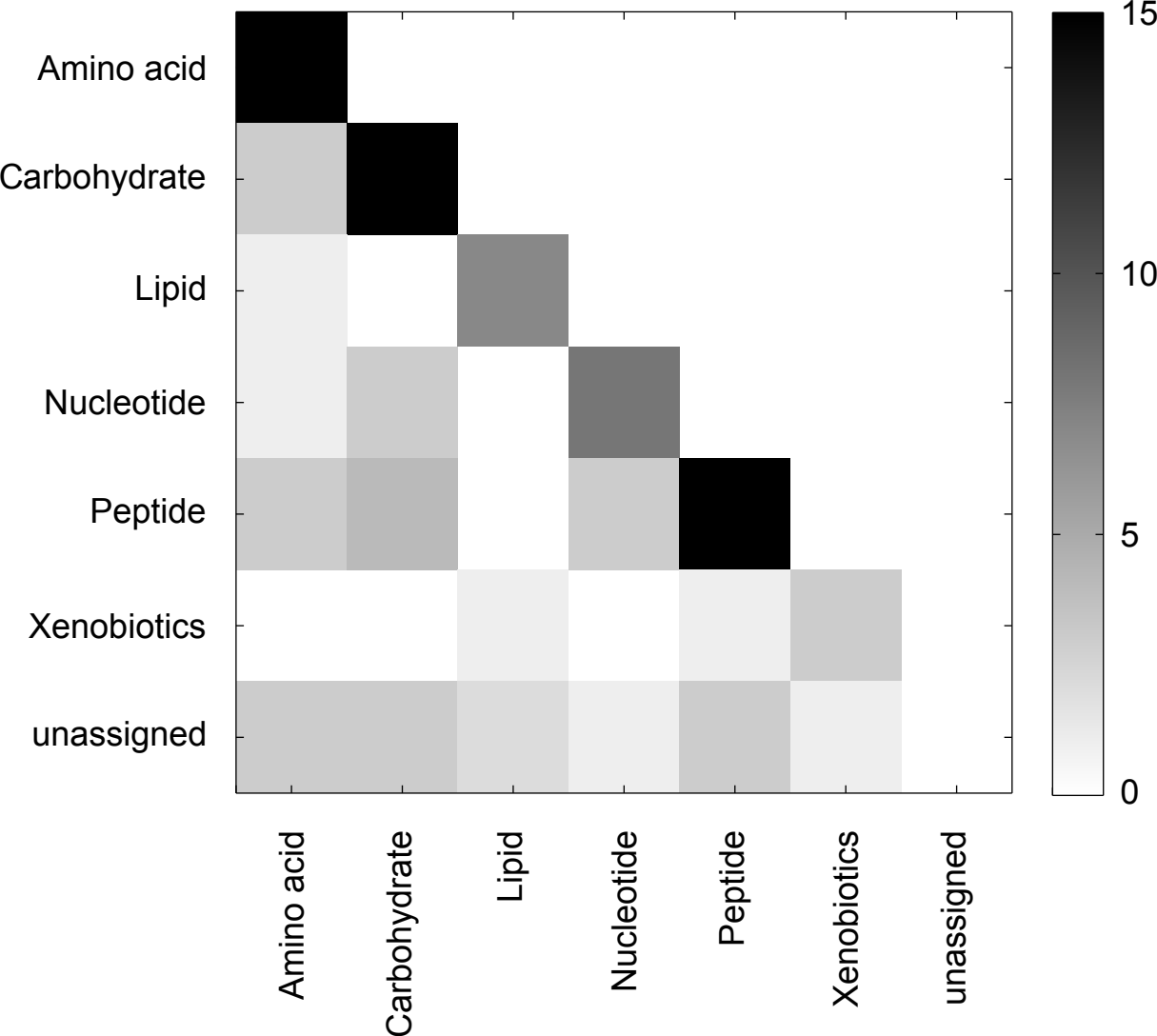

Supplement: S8 Fig — On this coarse level, only few but highly significant shared regulatory signatures are observable. Note that the color-scale is capped at 15, the maximum number of shared binding sites was 4 between carbohydrate and peptide metabolism. The category “unassigned” includes all unknown metabolites. (PDF) [file pgen.1005274.s009.pdf]

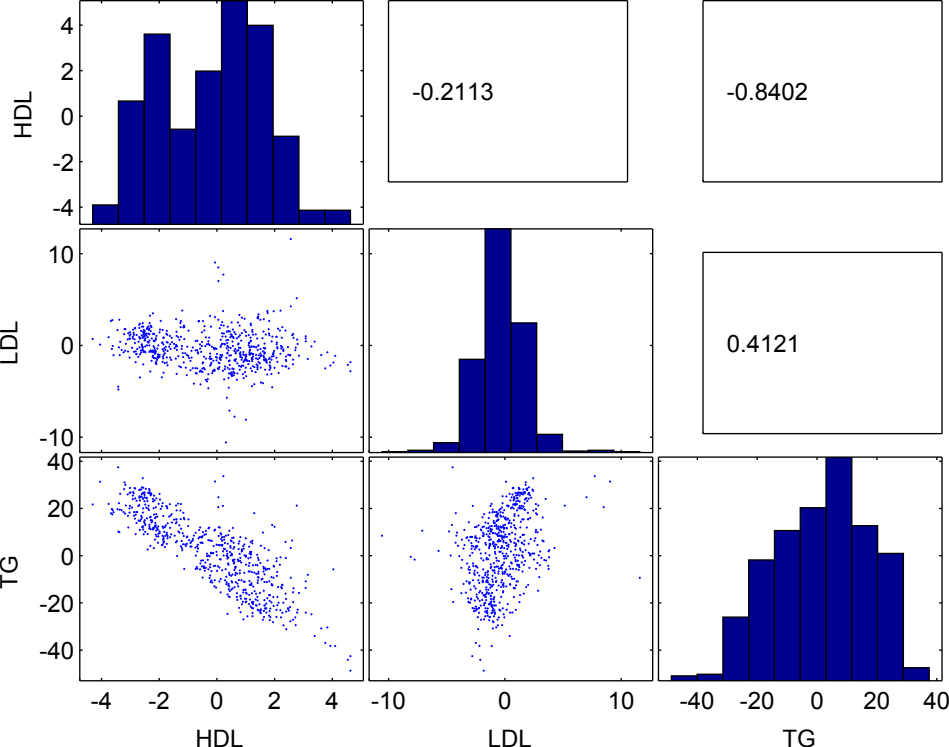

Supplement: S9 Fig — Comparison of beta values from linear regression analysis. Each dot represents either a transcript or a metabolite in the BMTI. We observe a strong anti-correlation between HDL and TG associations, modest positive correlation between TG and LDL and a very weak anti-correlation for HDL and LDL. (PDF) [file pgen.1005274.s010.pdf]

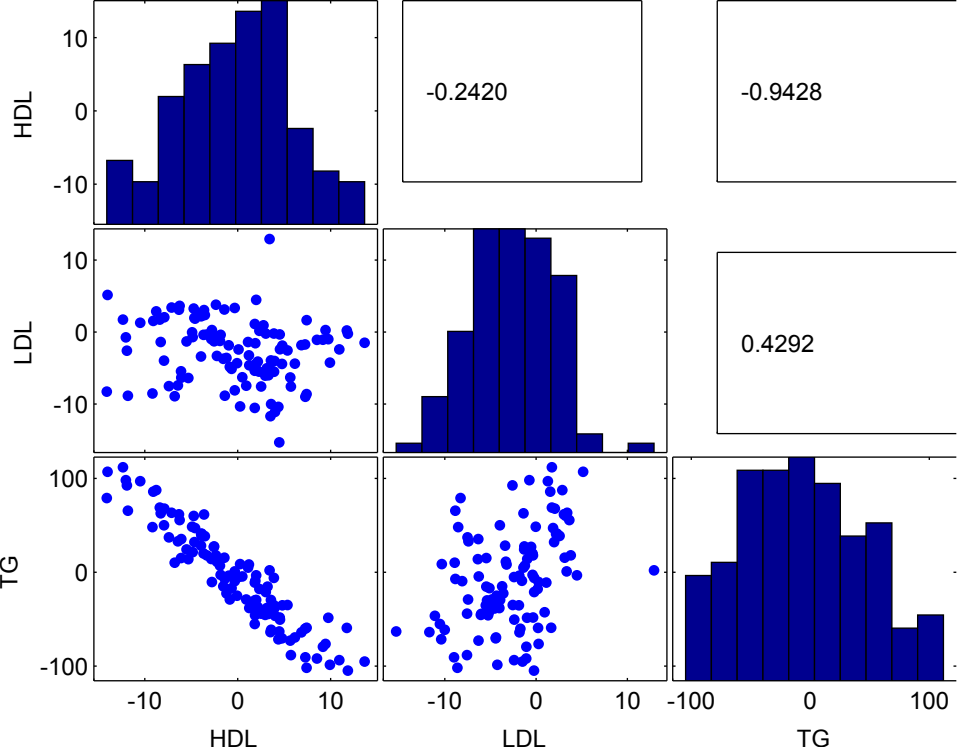

Supplement: S10 Fig — Comparison of beta values from linear regression analysis. Each dot represents either a GO term or a metabolic pathway in the PIN. Even more profound than for single transcripts and metabolites (S9 Fig), there is a strong anti-correlation between HDL and TG associations. The correlations for HDL and LDL as well as LDL and TG associations are comparable to the results from S9 Fig. (PDF) [file pgen.1005274.s011.pdf]

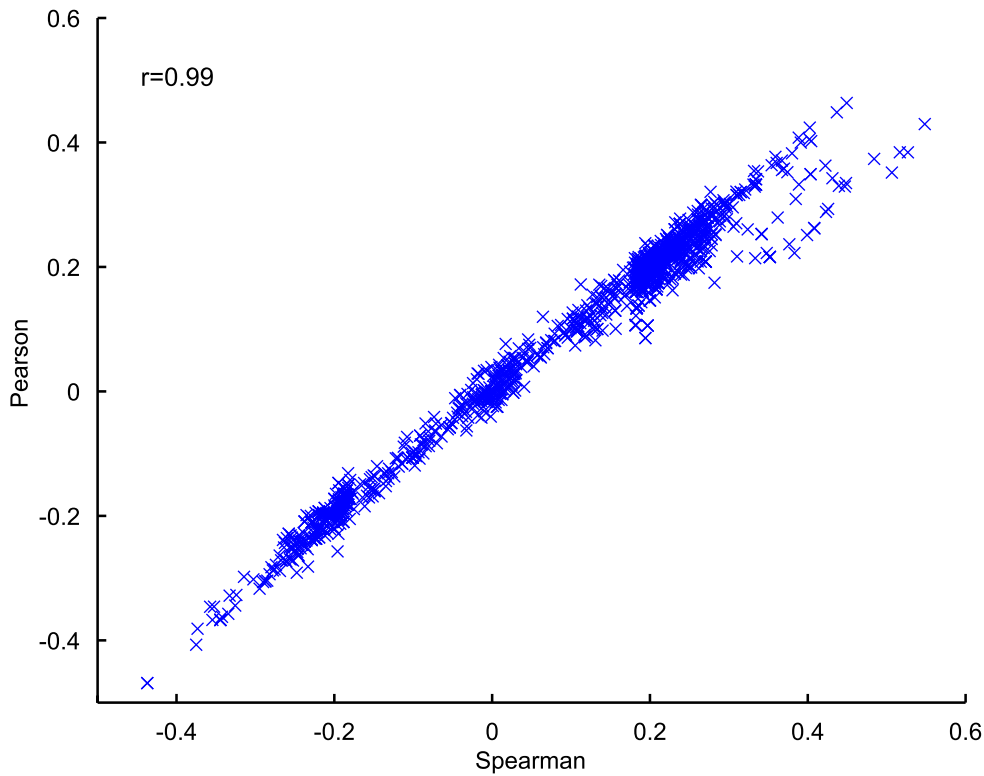

Supplement: S11 Fig — Similar to the results of transcripts and metabolites (S1 Fig), we observe high concordance of Spearman and Pearson correlation coefficients for the pathway scores (r = 0.99). (PDF) [file pgen.1005274.s012.pdf]
